# Operando Decoding Ion‐Conductive Switch in Stimuli‐Responsive Hydrogel by Nanodiamond‐Based Quantum Sensing

**DOI:** 10.1002/advs.202406944

**Published:** 2024-09-23

**Authors:** Ruqiang Dou, Zan Li, Guoli Zhu, Chao Lin, Frank X. Liu, Biao Wang

**Affiliations:** ^1^ Research Institute of Interdisciplinary Sciences & School of Materials Science and Engineering Dongguan University of Technology Dongguan 523808 China; ^2^ Department of Physics The Chinese University of Hong Kong Shatin, New Territories Hong Kong 999077 China; ^3^ Department of Mechanical and Aerospace Engineering Hong Kong University of Science and Technology Clear Water Bay, Kowloon Hong Kong 999077 China; ^4^ School of Physics and Sino‐French Institute of Nuclear Engineering and Technology Sun Yat‐sen University Guangzhou 510275 China

**Keywords:** Hydrogel, nanodiamond, PNIPAM‐AM, quantum sensing, sol‐gel transition

## Abstract

Thermal‐responsive hydrogels are developed as ion‐conductive switchs for energy storage devices, however, the molecule mechanism of switch on/off remains unclear. Here, poly(N‐isopropylacrylamide‐co‐acrylamide) hydrogel is synthesized as a model material and nanodiamond (ND) based quantum sensing for phase change study is developed. First, micro‐scale phase separation with cross‐linked mesh structure after sol‐gel transition is visualized in situ and water molecules are trapped by polymer chains and on a chemically “frozen” state. Then, the nano‐scale inhomogeneous distributions of viscosity, thermal conductivity and ionic mobility in hydrogel at high temperature are observed by measuring the rotation, translation and zero‐field splitting of NDs. Besides, the ionic mobility of hydrogel is found to be dependent not only on temperature but also on polymer concentration. These observations suggested that the physical “wall” induced by inhomogeneous phase separation at microscopic scale blocked the ion conduction pathways, providing a potential intrinsic explanation for ion migration shut‐down of ionic hydrogels at high temperature.

## Graphical abstract

The study develops nanodiamond (ND) based quantum sensing for operando decoding phase behavior at microscopic scale in stimuli‐responsive hydrogel. The rotation, translation and zero‐field splitting of NDs at different phases reveal the spatial distribution of viscoelastic and thermodynamic properties within 3D polymer networks, disclosing the inhomogeneous phase transition at nano‐scale and accounting for the ion migration shut‐down at high temperatures.

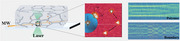

## 1 Introduction

Stimuli‐responsive hydrogels have attracted considerable interest as functional materials for drug delivery,^[^ ^1^ ^]^ advanced bioengineering,^[^ ^2^ ^]^ battery technology,^[^ ^3^ ^]^ smart electronic devices,^[^ ^4^ ^]^ etc. Among different stimulus, temperature has received significant attention due to its adaptability to various chemical/electrochemical devices and its ease of remote control to enhance device safety.^[^ ^5^ ^]^ Poly(N‐isopropylacrylamide‐co‐acrylamide) (PNIPAM‐AM) is a typical thermo‐responsive hydrogel, using N‐isopropylacrylamide and acrylamide as monomers. PNIPAM‐AM hydrogel (aqueous solution) exhibits a reversible sol‐gel phase transition upon increasing the temperature.^[^ ^3^, ^6^ ^]^ The hydrogel is on a freely flowing, transparent and ion‐conductive sol state when the temperature is below the lower critical solution temperature (LCST). However, surpassing the LCST leads to the transformation of the hydrogel into a stationary and opaque gel state,^[^ ^3a^ ^]^ on which the ion migration stops. Therefore, PNIPAM‐AM is an effective ion‐conductive switch and has been developed as the smart electrolyte to prevent ion migration at high temperatures for safety considerations, however, the fundamental factors influencing the temperature‐dependent phase behavior and ion conductivity remain unclear.^[^ ^5b^ ^]^

Numerous studies have explored the molecular mechanisms underlying the sol‐gel phase transition and the corresponding temperature‐dependent ion conductivity. While some investigations attribute this transition to osmotic pressures influenced by physical parameters like cross‐linking and charge localization, these physical parameters lack a molecular‐level understanding of the phase transition due to limited knowledge regarding the molecular reactions occurring within the polymer network.^[^ ^7^ ^]^ To address this limitation, a hydrogen bond model has been proposed.^[^ ^8^ ^]^ Breaking down of the hydrogen bond between the polymer chain and water molecules was found to trigger the phase transition of thermo‐responsive hydrogel.^[^ ^8b,d^ ^]^ The dynamics of hydrogen bond have been shown to exert significant control over the physical and chemical properties as well as the phase behavior of hydrogels.^[^ ^8f^ ^]^ However, the hydrogen bond model encounters challenges in explaining the temperature‐dependent ion conductivity, primarily arising from the limited information on hydrogel properties at the microscopic scale, such as local changes in viscoelasticity and thermal conductivity.

Generally, ion conductivity in hydrogel is strongly dependent on the local physical/chemical properties at nanoscale. Therefore, effective sensing on local properties evolution is important, and a number of sensing methods have been developed, such as nuclear magnetic resonance,^[^ ^9^ ^]^ laser scattering,^[^ ^8^, ^10^ ^]^ Fourier transform infrared spectroscopy^[^ ^8c,e^ ^]^ and Raman spectroscopy.^[^ ^8^, ^11^ ^]^ Although present sensing methods can provide the information of molecule vibration, the signal is averaged over a large sensing area and fail to disclose the nanoscale fluctuations. The visualization of local physical/chemical properties, such as the spatially resolved distribution of temperature, viscosity coefficient and ionic mobility at nano‐scale, are seldom reported in the literature. More comprehensive investigations are needed to understand the origin of temperature‐dependent ion conductivity in thermal‐responsive ionic hydrogels (e.g., PNIPAM‐AM).

Nanodiamonds (NDs) containing nitrogen vacancy (NV) centers have emerged as promising nanoscale quantum sensors.^[^ ^12^ ^]^ NDs possess exceptional characteristics, including robust photo‐resistance,^[^ ^13^ ^]^ strong chemical stability^[^ ^14^ ^]^ in extreme conditions, high thermal conductivity^[^ ^15^ ^]^ and nano‐scale spatial resolution. Therefore, NDs are adaptive to various systems, such as operando electronics, living cells and chemical reactions. Until present, ND‐based quantum sensors have been developed and applied in many research areas, such as mapping the temperature distribution in nano/micro electronic devices,^[^ ^16^ ^]^ local thermodynamics study in living cells,^[^ ^12^, ^17^ ^]^ nano‐scale magnetic studies,^[^ ^18^ ^]^ high precision electric field sensing,^[^ ^19^ ^]^ local mechanical deformation reconstruction of 2D materials,^[^ ^20^ ^]^ and operando decoding chemical events in solutions.^[^ ^21^ ^]^ Optically detected magnetic resonance (ODMR) is a common approach to decode nanoscale physical parameters recorded by individual NDs.^[^ ^22^ ^]^ ODMR is an optical method which is non‐invasive and thus has little effect on the systems or ongoing reactions. Previous studies have showed that the hydrogen bonding dynamics, including the hydrogen bond evolution, comparison and transition in different hydrogen bonding configurations regulated the sol‐gel transition of hydrogel.^[^ ^8b,f^ ^]^ The discrete ND particles in hydrogel are hard to form hydrogen bond networks, though the ─OH group is usually dominated on the ND surface.^[^ ^23^ ^]^ Therefore, the addition of a small amount of NDs in hydrogel has little influence on phase change behaviors. These unique advantages make ND an ideal sensing method for operando decoding the local physical/chemical properties evolution in sol‐gel phase transition of thermo‐responsive ionic hydrogels.

In this study, we developed an ND based quantum sensing to decode the phase change behaviors in stimuli responsive hydrogels in situ. The sensing performance was demonstrated by investigating the molecule machinery of ion‐conductive switch in PNIPAM‐AM hydrogel, a typical thermal responsive ionic hydrogel electrolyte with the LCST of 45 °C, aiming to investigate the phase change behaviors along with sol‐gel transition and provide valuable insights into the design of functional ionic hydrogels for energy storage devices. First, the sol‐gel transition and the corresponding phase separation were visualized by in situ Raman microscopy combined with confocal microscope. Phase separation after sol‐gel transition was visualized and each phase was identified. The hydrogen bonding dynamics in different phases and the chemical states of water molecules were studied. Then, the viscoelastic and thermodynamic properties in hydrogel before and after sol‐gel transition were investigated. The particle migration in PNIPAM‐AM hydrogel at different temperatures was studied by measuring the rotation and translation information of NDs. The NDs motion performance and spatial distribution were analyzed, disclosing the non‐uniform distribution of viscoelasticity. Besides, the local temperature difference at nano‐scale before and after sol‐gel transition were investigated with the nonuniformity of heat dissipation pathways visualized. The inhomogeneous thermodynamic and viscoelastic properties at nano‐scale provided a possible intrinsic explanation for ion migration shut‐down at high temperature in PNIPAM‐AM hydrogel. Finally, a model of ion migration in hydrogel at different temperatures was constructed. The model was verified by monitoring the resistance evolution of a resistor with hydrogel at different concentrations as the electrolyte, contributing to explain the molecule mechanism of ion‐conductive switch in thermal responsive hydrogel. This study presents a powerful quantum sensor for probing nano‐scale phase change behaviors of stimuli‐responsive ionic hydrogels, providing valuable insights into the origin of temperature dependent ion conductivity along with sol‐gel phase transition.

## 2 Results and Discussion

### 2.1 Visualization of Phase Separation in PNIPAM‐AM Hydrogel

**Figure 1:**
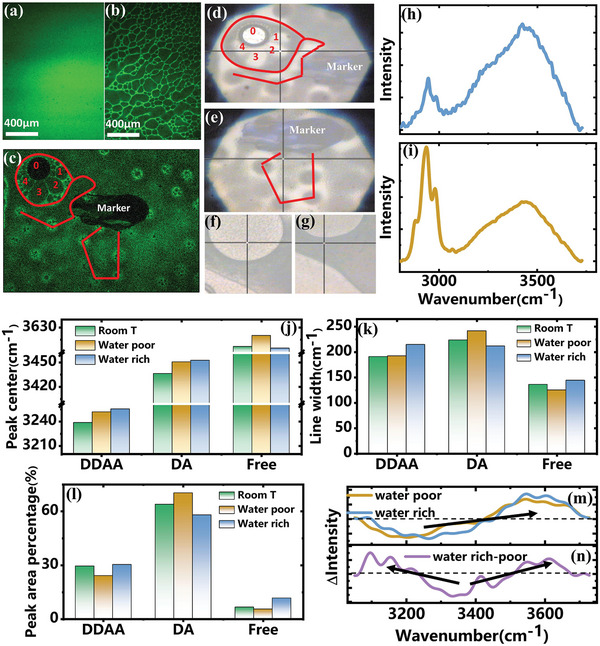
Visualization of phase change in sol‐gel transition and the corresponding hydrogen bonding dynamics in this process. Fluorescence images of FITC contained hydrogel a) before and b) after sol‐gel transition. Correlation of c) fluorescence images in confocal microscope and d,e) optical photos in camera equipped Raman spectrometer. A f) bright and g) dark regions were selected and h,i) the corresponding Raman spectra for (f) and (g), respectively. ─OH stretching vibration analysis of hydrogel at room temperature and ≈60 °C after sol‐gel transition, using three Gaussian components to fit the Raman spectra. The j) peak center, k) linewidth and l) peak area percentage of the three components in different phases in this process. The differential phonon spectra of m) water rich and poor phases with respect to Raman spectrum at room temperature, and n) water rich phase with respect to water poor phase.

To investigate the change in ion migration of PNIPAM‐AM hydrogel, the physical and chemical properties during sol‐gel phase transition were first studied. The fluorescence images of FITC contained hydrogel at room temperature and ≈60 °C were shown in **Figure** **1**a,b, respectively. At room temperature (25 °C), the distribution of fluorescence intensity was homogeneous, indicating the uniform distribution of polymer chains in water medium (Figure 1a). However, a dark region and a bright region with a cross‐linked mesh structure were observed after sol‐gel phase transition (≈60 °C) in Figure 1b, suggesting the occurrence of phase separation when the polymer was on gel state. The phase separation was usually accompanied with a series of “island” (Figure S1a, Supporting Information). In each of the “island”, the fine structure was also cross‐linked mesh shape (Figure S1b, Supporting Information). As the hydrogel was confined in a ≈100 µm layer in between two cover glass (see Experimental Section), a phase separation with a zigzag form was observed in vertical direction (z focus direction or perpendicular to the cover glass plane) in Figure S1c (Supporting Information) and FITC rich phase was prone to distribute near cover glass. The phase separation disappeared and recovered to be homogeneous in fluorescence intensity when cooling down (Figure S1d and Video S1, Supporting Information).

To correlate the FITC rich/poor phase with polymer chains/water molecules, in situ Raman microscopy was used to characterize the hydrogel. A marker was introduced to the hydrogel and a cross‐linked mesh structure accompanied with small islands after sol‐gel transition were observed in Figure 1c. Then the sample was transferred to in situ Raman microscopy. The marker was located, and the photos taken by the camera equipped in the microscope were presented in Figure 1d,e, respectively. Similarly, the phase separation was observed. By comparing the photos with the confocal fluorescence image (indicated by the red lines in Figure 1c–e), we concluded that the FITC rich and poor phases were corresponding to dark and bright phases in Raman microscope, respectively (the merged figures could be found in Figure S2, Supporting Information). A bright and dark region (the black cross in Figure 1f,g, respectively) were selected, and the corresponding Raman spectrum could be found in Figure 1h,i, respectively. Compared with the Raman spectra of pure water and hydrogel powder (Figure S3, Supporting Information), the higher intensity in 3200–3600 cm^−1^ in Figure 1h indicated that water molecules were rich in the measured region (bright region in Figure 1f), which corresponded to FITC poor phase. Similarly, the intensity in 3200–3600 cm^−1^ in Figure 1i was lower, suggesting a water poor phase (polymer rich), which corresponded to the dark region in Figure 1g and FITC rich region. Consistent results were observed and confirmed this correlation by changing different regions (Figure S4, Supporting Information). The observations disclosed that the water molecules after sol‐gel transition were confined in a small region formed by 3D polymer networks with size ranging from hundreds of nanometers to hundreds of micrometers.

To investigate the chemical state of water molecules and associated HB dynamics, Raman spectra in the range of 3050–3800 cm^−1^ were analyzed. As illustrated in Figure S5 (Supporting Information), the peaks at ≈3620, 3420, 3210 cm^−1^ were attributed to −OH stretching vibration with no HB regulation (Free), asymmetrical HB regulation (DA) and symmetrical HB regulation (DDAA),^[^ ^24^ ^]^ respectively. The Raman spectra of hydrogel at room temperature and ≈60 °C (water rich and poor phases) were fitted by three Gaussian components with the raw data shown in Figures S6–S8 (Supporting Information), respectively. The peak center of DDAA and DA in both water rich and poor phases at high temperature was larger than that at room temperature in Figure 1j, suggesting the enhanced −OH chemical bond. This could be originated from the temperature effect on −OH stretching vibration. As presented in Figure 1k, in water rich phase, the linewidth of DDAA became much larger while that of DA was obviously smaller, compared to the linewidth of Raman spectra at room temperature, indicating the enhanced hydrogen bond between individual water molecules and the line structure of water molecules in water rich phase became weak. As a comparison, the linewidth of DA in water poor phase was relatively larger while that of DDAA had little change, suggesting that the enhanced line structure of water molecules in water poor phase. The peak area percentage in Figure 1l suggested that hydrogen bonding structure transformed from DDAA to DA in water poor phase, while more free water molecules occurred in water rich phase. Blue shift could be found in the differential phonon spectrum with respect to Raman spectrum at room temperature, suggesting the enhanced −OH chemical bond and decreased hydrogen bond between water–water molecules (Figure 1m). When comparing the Raman spectrum of water rich and poor phases (Figure 1n), the transition from DA to DDAA and free water molecules could be observed (the normalized Raman spectrum could be found in Figure S9, Supporting Information). These observations indicated that water molecules might be prone to form clusters with DDAA structure in water rich phase. Free water molecules could exist in the clusters. However, line structure (DA) might have more opportunities to exist in polymer rich (water poor) phase.

### 2.2 Viscoelastic and Thermodynamic Properties in Sol‐gel Phase Transition

**Figure 2:**
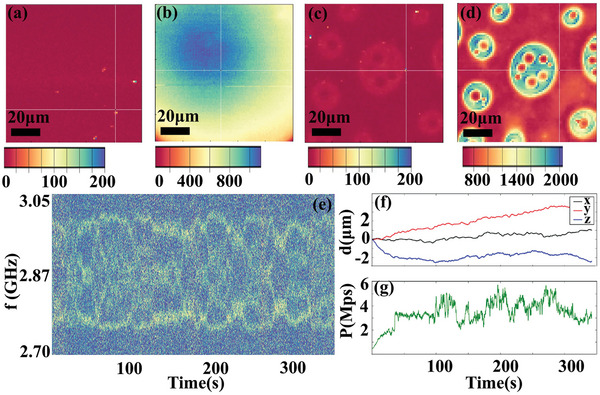
Viscoelastic properties of hydrogel at room temperature. Fluorescence image of a) NDs, b) FITC of hydrogel at room temperature, and c,d) at ≈60 °C, taken by a home‐built confocal microscope with two excitation and data collection pathways. e) The ODMR spectra under applied magnetic field as a function of time of a representative ND in hydrogel at room temperature. The contrast of each ODMR spectrum is encoded in color. f) The 3D translation and g) the photon count rate as a function of time of this ND in the same process.

The phase transition in hydrogel typically started from the changes in local mechanic and thermal properties at nano‐scale. Thus, NDs were introduced to decode the viscoelastic and thermodynamic information after differentiation of water rich/poor phase in sol‐gel transition. To decouple the fluorescence of NDs and FITC, a home‐built confocal microscope with two excitation and collection pathways (473 and 589 nm, see Experimental Section) was adopted to characterize the ND and FITC contained hydrogel. **Figure** **2**a showed the NDs fluorescence image with the ND distribution at different focus shown in Figure S10 (Supporting Information). NDs randomly distributed in the hydrogel at room temperature. The corresponding fluorescence image of FITC in the same region was presented in Figure 2b. The concentration of ND was relatively lower than that of polymer with only several NDs found in the field of view, thus the hydrogen bonding interaction was dominated between water molecules and polymer chains. The contribution from ─OH group on the ND surface was small. The difference in fluorescence intensity within the field of view was small (Figure 2b), suggesting that the phase separation was not obvious at room temperature. As a comparison, when the temperature was increased to ≈60 °C, visualization of NDs maintained (Figure 2c), while the uniformity in the distribution of FITC fluorescence intensity significantly changed (Figure 2d). Phase separation with cross‐linked “island” structure occurred (Figure 2c,d), which was similar to the observation in commercial confocal microscope. Based on the correlation established in last section, the bright (FITC rich) and dark (FITC poor) region was corresponded to water poor and rich phase, respectively. The NDs might occur in each phase or the boundary at the interface (Figure 2c), enabling local properties detection in sol‐gel phase transition of hydrogel. One should note that the scale (colour) bars in the fluorescence images (Figure 2a–d) were not consistent, mainly originated from the large difference in fluorescence intensity of NDs and FITC.

The viscoelastic properties of hydrogel at room temperature were firstly investigated. Figure 2e showed the ODMR spectra under applied magnetic field as a function of time of a representative ND in hydrogel at room temperature. The resonance frequencies varied significantly with time and fluctuated largely and rapidly, suggesting a large‐scale rotation of the ND in hydrogel at room temperature. In extreme cases, the resonance frequencies fluctuated too fast, leading to the mixing of ODMR peaks (Figure S11, Supporting Information). The translation of this ND in the same duration time could be simultaneously recorded and presented in Figure 2f. A few micrometers displacement in 350 s could be observed. The corresponding counts fluctuations could be found in Figure 2g. The photon counts always fluctuated in a reasonable range, suggesting that this ND could be tracked during the recording time. As the tracking limit was ≈1 µm s^−1^, the above observations suggested that the hydrogel at room temperature had a moderate viscosity. Although it was significantly higher than that of pure water (we could hardly track any ND in pure water), particles with the size of few tens to several hundred of nanometers could migrate in the hydrogel at room temperature.

**Figure 3:**
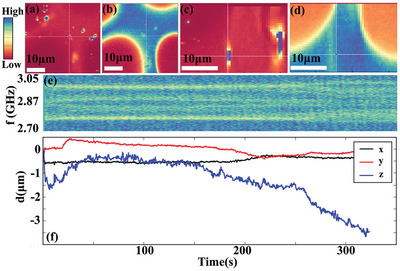
Translation and rotation tracking of a nanodiamond in water poor phase of the hydrogel after sol‐gel transition at ≈60 °C. Fluorescence images for determination of ND location taken by home‐built confocal microscope. The *xy* plane scanning fluorescence of a) NDs and b) the corresponding FITC. The fluorescence of *xz* scanning of c) NDs and d) the corresponding FITC for reconstruction of ND location. e) The ODMR spectra under applied magnetic field as a function of time, f) the corresponding translation of this ND in the same process. The color from red to blue in (a–e) represents from the lowest to highest value.

Then, the viscoelastic properties in different phases of hydrogel after sol‐gel transition were studied. **Figure** **3**a showed the confocal image of NDs with *xy* plane scanning (*xyz* frame could be found in Experimental Section). An ND was marked by white cross. The corresponding image of FITC could be found in Figure 3b. The marked ND was in water poor phase in *xy* plane. Similarly, the confocal images of this ND and FITC with *xz* plane scanning were presented in Figure 3c,d, respectively. The ND was found to be in the water poor phase in *xz* plane. Then the exact location of the ND could be reconstructed, and it was located inner of water poor phase. Figure 3e showed the ODMR spectra of the ND (in water poor phase) as a function of time under applied magnetic field. Peak splitting could also be observed (Figure S12, Supporting Information). The time‐dependent ODMR spectra of the ND showed some difference compared to that at room temperature. The resonance frequency fluctuation slowed down and the rapid changes even disappeared, suggesting that the ND had little rotation in water poor phase. The corresponding translation in *xyz* direction was shown in Figure 3f. The location shift in both *x* and *y* direction was within 0.5 µm. The large‐scale displacement in *z* direction was mainly due to thermal drift, leading to focus change (Figure S13, Supporting Information). Similar result was observed in all NDs located in water poor phase with another example shown in Figure S14 (Supporting Information). The observations disclosed that ND in water poor phase was almost in a fixed state with little rotation and translation, indicating a significant enhancement in the viscosity in water poor phase.

**Figure 4:**
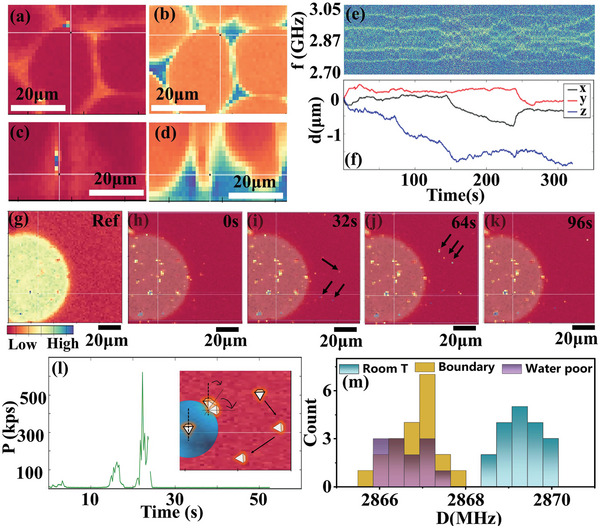
Translation, rotation and thermodynamics tracking of NDs in water rich phase and the boundary of the water poor/rich phase of the hydrogel after sol‐gel transition at ≈60 °C. Fluorescence images for determination of ND location taken by home‐built confocal microscope. The *xy* plane scanning fluorescence of a) NDs and b) the corresponding FITC. The fluorescence of *xz* scanning of c) NDs and d) the corresponding FITC to determine the location of the marked ND. e) The ODMR spectra under applied magnetic field as a function of time, f) the corresponding translation of this ND. g) A selected fluorescence image with the field of view showing the water rich and poor phase, a time lapse images of NDs distribution in this region at h) 0, i) 32, j) 64 and k) 96 s. l) Tracking the counts rate in water rich phase, with the inset showing the schematic of NDs rotation and translation at different phases in hydrogel after sol‐gel transition. m) The histogram of zero field splitting *D* of individual NDs in hydrogel at room temperature, in water poor phase and at the boundary of water poor/rich phase at ≈60 °C. The color from red to blue in (a–e and g–k) represents intensity from the lowest to highest value.

Similarly, the rotation and translation of ND at the boundary of water rich and poor phases were studied. The fluorescence images of ND and FITC with *xy* (**Figure** **4**a,b) and *xz* plane scanning (Figure 4c,d) reconstructed the geometrical configuration of the ND, showing that this ND located at the interface of the two phases. The time dependent ODMR spectra could be found in Figure 4e. The resonance frequencies had small fluctuations, suggesting the mild rotation of the ND at the interface. The rotations of more NDs were analyzed, and it was found that NDs at the boundary of water rich and poor phases had a moderate or even little rotation (Figure S15, Supporting Information). Figure 4f presented the translation of the ND. Similar to what was observed in water poor phase, the ND had a very small translation (*xy* direction: <1 µm in 300 s, *z* displacement mainly came from thermal drift), suggesting the almost fixed location of ND at the interface of the two phases.

Figure 4g showed the confocal image of a circular‐shape water poor phase with the time dependent ND distribution in the region presented in Figure 4h‐k, showing the ND translation in different phases. The relative location of NDs in water poor phase (bright region) or at the boundary had little change (Figure 4i‐k vs h). As a comparison, NDs moved fast in water rich phase, and they might quickly occur or disappear in the field of view (black arrows in Figure 4i,j, more data could be found in Figure S16 and Video S2, Supporting Information). Due to the fast translation, the NDs in water rich phase were hardly to be continuously tracked (Figure S17, Supporting Information). As indicated by the large‐scale photon counts fluctuations in Figure 4l, even though an ND was occasionally tracked by the laser with a considerable photon counts rate, it might fast escape out of our tracking window. As our tracking limit was ≈1 µm s^−1^, the translation speed of ND in water rich phase might be a few or even tens of micrometer per second, which was similar to the Brownian motion in pure water. As illustrated in the inset in Figure 4l, NDs in water poor phase were in a fixed state, while those in water rich phase could “freely” migrate. NDs “attached” on the interface of the two phases were most likely in an intermediate state, waving slightly with little change in the location. In addition, NDs in water rich phase might approach to the water poor phase, however, they could hardly break the interface and transfer into the water poor phase (Figure S18 and Video S3, Supporting Information). The water poor phase was prone to appear near the upper or lower substrate (Figure S19, Supporting Information) and might surround the water rich phase, suggesting the inhomogeneous phase separation.

The local temperature difference in hydrogel at room temperature and ≈60 °C recorded by individual NDs under no magnetic field was also analyzed with examples of ODMR spectra shown in Figure S20 (Supporting Information). As presented in Figure 4m, at room temperature, the distribution of zero field splitting (*D*) was in Gaussian shape with the average value of 2869.34 MHz (Figure S21a, Supporting Information), suggesting that the difference in *D* was mainly due to the measurement uncertainty. As a comparison, the averaged *D* shift of NDs after sol‐gel transition was 2866.75 MHz (Figure S21b,c, Supporting Information), resulting in an averaged local temperature of 59.6 °C, which was consistent with the set temperature measured by a standard thermometer (≈60 °C). However, the statistics in *D* distribution at high temperatures were non‐Gaussian shape, suggesting the occurrence of local temperature difference which was beyond the measurement uncertainty. In addition, the standard deviation of local temperatures in water poor phase was significantly smaller than that at the boundary, probably originated from the fast heat transference with water rich phase when the NDs were located at the interface of the two phases.

The observations disclosed that the viscoelastic and thermodynamic properties had a significant change when temperature increasing. The inhomogeneous spatial distribution of viscosity and local temperature at nano‐scale occurred after sol‐gel transition. Although the water molecules were in a confined state (Figure 1j‐n), the physical and chemical activities were still high, enabling the free particle rotation and translation in water rich phase after sol‐gel transition. This indicated that the confined or frozen state of water molecules was not a major contributor of shut down of ion migration in hydrogel at high temperatures. Water poor phase acted as the “wall” in the hydrogel mixture after sol‐gel transition, that inhibited the particles transference. In addition, it might attach onto the substrates (e.g., electrodes) and surround the water rich phase. The lacking of effective conduction pathways might be a possible reason of shut down in the ion migration after sol‐gel transition when used as electrolyte. This was not an intrinsic property of hydrogel, thus the effective shut down of ion migration at high temperatures required a specific concentration of hydrogel aqueous solution.

### 2.3 Electrical Resistance Dependent on Ion‐Conductive “Wall”

**Figure 5:**
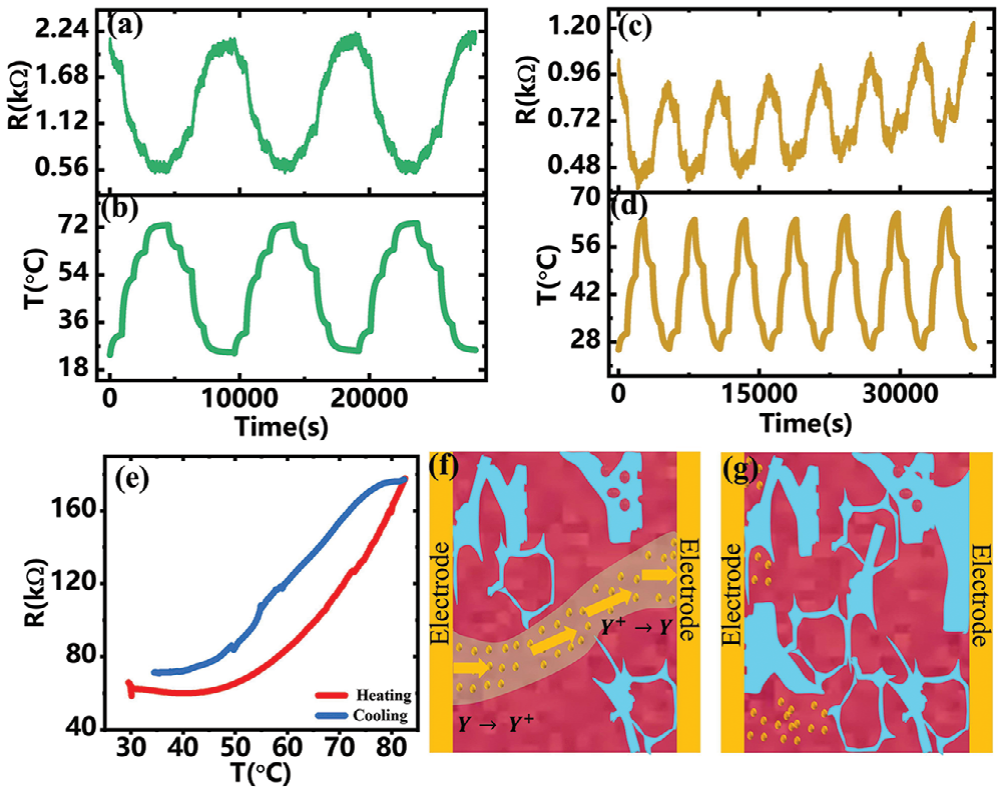
Dynamics in the electrical resistance of a resistor with the hydrogel as electrolyte. a) Resistance as a function of time when the 0.1 m ZnSO_4_ was used as the electrolyte. b) The corresponding temperature evolution with time in the same process. c) The resistance and d) temperature as a function of time when the hydrogel was added into the electrolyte with a mass ratio of 5%. e) Resistance as a function of temperature when the hydrogel concentration in electrolyte was improved to 20%. The red and blue line represented the heating and cooling process. A schematic showing the ion‐conductive pathways after sol‐gel transition when the hydrogel concentration in electrolyte was (f) low and (g) high.

To verify the inference of concentration dependent ion‐conductive switch, a specially designed resistor was fabricated with the schematic and a phot of the device shown in Figure S22a,b (Supporting Information), respectively. As a reference, 0.1 m ZnSO_4_ was selected as the electrolyte. The resistance of the resistor as a function of time was shown in **Figure** **5**a with the corresponding temperature evolution with time in the same process was presented in Figure 5b. A negative relationship between resistance and temperature with good reversibility could be observed, mainly due to the faster ion motion speed upon increasing the temperature. A similar result could be observed when a small amount of hydrogel was added (mass ratio: 5%) in Figure 5c,d. Differently, the envelope of resistance‐time curve had an upward trend, suggesting that the resistance reversibility in a heating and cooling cycle (sol→gel→sol) was not as good as that of pure ZnSO_4_ solution. This might be induced by inhomogeneous phase transition at microscopy scale, which was consistent with the fluorescence images in confocal microscope (the details were discussed in Figure S23, Supporting Information). In contrast, a positive temperature coefficient in the relationship of resistance and temperature was observed when the hydrogel mass ratio was improved to 20% (Figure 5e). The ion thermal motion was suppressed, and electrolyte resistance increased under heating stimulation, suggesting that the ion conduction was significantly deteriorated in sol‐gel transition process.

The observations suggested a concentration threshold for an ion conductive switch with the PNIPAM‐AM hydrogel as the electrolyte (details could be found in Figure S24, Supporting Information). A schematic showing the concentration dependent ion‐conductive switch was presented in Figure 5f,g. When the hydrogel concentration was low, the ion‐conductive switch does not work, because the water poor phase after sol‐gel transition could not completely block ion transference, due to the existing of ion‐conductive pathways (Figure 5f). In contrast, water poor phase would almost cover the whole electrode surface at high hydrogel concentrations (Figure S19, Supporting Information). The hydrogel after sol‐gel transition had a high degree of cross‐linking, that totally blocked the ion‐conductive pathways, resulting in the shut down of the switch at the temperature above LCST (Figure 5g).

## 3 Conclusion

In summary, we established an in situ method for decoding the phase change behaviours in stimuli responsive hydrogels, specifically the ion‐conductive switch (PNIPAM‐AM), using nanodiamond based quantum sensing. Firstly, visualization of micro‐scale phase separation in situ was achieved by Raman microscopy combined with FITC‐labelling fluorescence imaging, disclosing cross‐linked mesh structure with water‐rich/poor phases identified at the temperature above LCST. Analysis of Raman spectra revealed transitions between different hydrogen bonding configurations, resulting in a chemically confined state of water molecules in hydrogel networks. Then, the non‐uniform distribution in hydrogel viscosity at nano‐scale after sol‐gel transition was found by monitoring the translation and rotation of NDs in different phases. In addition, nano‐scale local temperature variations were commonly detected in hydrogel post‐transition, indicating the inhomogeneous heat dissipation pathways. By correlating the timely and spatially resolved viscoelastic and thermodynamic characteristics with microstructure of hydrogel, the water‐poor phase was found to largely influence the ion mobility at high temperatures. This was supported by resistance‐temperature assessments, showing that an effective ion‐conductive switch required a specific hydrogel concentration. The observations suggested that the physical barriers induced by inhomogeneous phase separation at nano‐scale might be a possible intrinsic reason for temperature dependent ion conductivity of PNIPAM‐AM hydrogel.

Although PNIPAM‐AM hydrogel was used as a model material in this work, the application of ND based quantum sensing was not limited in this specific material. This technique could be extended to investigate the nano‐scale phase behaviors of various stimuli‐responsive ionic hydrogels (e.g., pH sensitive hydrogel), providing more information on ion/particle transference and molecular interactions within the hydrogel 3D networks. Such knowledge was important to design hydrogel based ion‐conductive switch, targeted medicine delivery system, tissue engineering device, etc.

## 4 Experimental Section

### Materials

The NDs with NV ensemble were purchased from NaBond Technologies. Each diamond nanocrystal contains ≈1600 NV centers. The NDs were dispersed in ethanol solution at a concentration of 10 μg mL^−1^, resulting in that the functional group of ─OH was dominated on the surface of NDs.^[^ ^21^, ^23^ ^]^ The TEM images of NDs could be found in Figure S25 (Supporting Information). The NDs had an irregular shape with a size distribution of a few tens to several hundred nanometers. The present size enabled the submicron spatial resolution when ND based sensing method was adopted. The zeta potential of ND sample suggested that the NDs were negatively charged (Figure S26, Supporting Information). Acrylamide (AM,≥99%), N‐isopropyl acrylamide (NIPAM,≥99%), Methylene‐bis‐acrylamide (MBAA,99%), potassium persulfate (KPS,99.99%), fluorescein 5‐isothiocyanate (FITC,90%), dimethylformamide (DMF,≥99.8%) and acetone (≥99%) were purchased from Sigma–Aldrich. All regents were used after purchase without further purification. Deionized water was produced by a HITECH laboratory water purification system with a resistivity of 18.2 MΩ·cm.

### Synthesis of PNIPAM‐AM

PNIPAM‐AM hydrogel with the molar ratio of NIPAM:AM ≈4:1 was synthesized using a free radical polymerization method. NIPAM (2.0 g), AM (0.315 g) and MBAA (0.224 g) were mixed with 25 mL DMF in a three‐neck flask. The mixture was stirred at 70 °C under a nitrogen atmosphere for 0.5 h. KPS of 0.033 g was then added as an initiator, and the reaction continued for 8 h at 70 °C with stirring at 300 r min^−1^. The reaction was stopped by cooling the mixture in ice water. The resulting mixture was precipitated with acetone, and the precipitate was placed in a Teflon pan. The copolymer was obtained by evaporating the remaining DMF and acetone in a vacuum drying oven at 50 °C for 12 h. The LCST of the synthesized PNIPAM‐AM at the concentration of 0.2 g mL^−1^ in water was ≈45 °C. The test temperature in the present experiment was 60 °C, aiming to ensure that almost all the hydrogel had become opaque gel state (the details could be found in Figure S27, Supporting Information).

### Visualization of Phase Separation

FITC water solution of 2 µL at the concentration of 25 mg mL^−1^ was added to 2 mL PNIPAM‐AM water solution with a concentration of 0.2 g mL^−1^. The mixture was sonicated for 10 min and then kept in the dark for 4 h to ensure well dispersion of FITC molecules in the copolymer environment. To visualize the phase separation, the mixture (30 µL) was placed between two cover glasses and sealed with epoxy resin, as depicted in Figure S28 (Supporting Information). The temperature was controlled using a heating ceramic. A Leica TCS SP8 X confocal microscope equipped with a 473 nm excitation laser was used to observe the phase separation.

### Raman Measurement

Raman spectra were measured using a Renishaw inVia commercial confocal Raman spectrometer with a spectral resolution of 1 cm^−1^. A backscattering geometry was realized by using the 50x long working distance objective of an Olympus BX40 microscope. The sample was excited with a 532 nm laser (Cobalt Samba 300), and the signal was detected by a thermoelectric cooled charge‐coupled array detector (iVac 316 CCD) operating at −70 °C. Raman spectra were recorded in the wavenumber range of 50–4000 cm^−1^. LED light was used to take the video or photos along with the Raman measurements.

### Differential Phonon Spectra

The Raman spectrum ranging from 3050 to 3800 cm^−1^ was selected for analysis. To eliminate the potential influence of the ─CH stretching vibration on the ─OH stretching vibration, the baseline was subtracted using an exponential function. After subtracting the baseline, the data was integrated to calculate an area value (referred to as A). The normalized Raman spectrum was then obtained by dividing the photon counts by A at each wavenumber. The normalized Raman spectrum of reference (e.g., pure water) was represented as *S_0_*, while *S(x)* represented the normalized Raman spectrum when the experimental conditions were altered (e.g., addition of PNIPAM‐AM or increasing temperature). The differential phonon spectra of the ─OH stretching vibration were obtained by subtracting *S_0_* from *S(x)*.

### ODMR Measurement

The temperature sensing of NDs was performed using continuous‐wave optically detected magnetic resonance (ODMR) with a home‐built confocal microscope, as illustrated in Figure S29 (Supporting Information). To pump the NV center spins, a 589 nm laser was utilized, while a 473 nm laser was used to excite the fluorescence dye (FITC). The spin states of the NV centers were manipulated by applying microwave through a copper wire. The fluorescence emitted by the NVs was collected using an avalanche photodiode (APD: SPCM‐AQRH‐15‐FC, Excelitas). The measured ODMR spectrum of the ND sensor was normalized to the counts of the off‐resonance signal. Then, the normalized ODMR spectrum was fitted by multi‐peak Lorentzian function in a nonlinear least‐square method.

### Device for ODMR Measurement

The device that enabled simultaneous ND based quantum sensing and hydrogel phase transition was illustrated in Figure S30 (Supporting Information). The cell was built onto a printed circuit board (PCB) with a piece of pre‐glued cover glass as the substrate. Cu wire (≈25 µm in diameter, Sigma–Aldrich) was used for microwave introduction. ND‐hydrogel mixture with the ND concentration of 10 µg mL^−1^ was dropped on the substrate. Then, another cover glass was put over the ND‐hydrogel mixture. The edge was sealed by epoxy resin to avoid water evaporation when temperature increasing. A heating ceramic was placed on top of the upper cover glass, allowing heating to be applied during ODMR recording. It should be noted that if the NDs have severe aggregation in the device, it would affect the sol‐gel transition of hydrogel, and the ODMR measurement could also be influenced. Thus, the ND concentration and dropping of the ND contained hydrogel should be finely controlled in each experiment to avoid ND aggregation.

### Preparation of ND Contained Hydrogel Electrolyte

The purchased ND (1 mg mL^−1^ in water) was diluted in deionized water to obtain the ND contained water solution at the concentration of 10 µg mL^−1^. ND contained ZnSO4 (0.1 m) electrolyte was prepared by mixing 40 mL of ND contained water (10 µg mL^−1^) and 1.150 g of 𝑍𝑛𝑆𝑂4 ∙ 7𝐻2𝑂 (Sigma–Aldrich), and sonicated for 10 min. The dried PNIPAM‐AM powder of 1 g was added into ZnSO4 electrolyte of 5 mL to obtain the hydrogel electrolyte of 0.2 g mL^−1^. The mixture was sealed and sonicated for 30 min, and kept in drying oven at 25 °C for 3 h. Repeat the sonication and stewing process for three times to make the polymer chains well dispersed in the electrolyte. Before each experiment, the hydrogel electrolyte was sonicated for 30 min to avoid the NDs aggregation. Similar process was conducted to obtain ND contained hydrogel electrolyte with various concentrations (e.g., 0.1 g mL^−1^).

### Decoding Local Properties in Hydrogel

**Figure 6:**
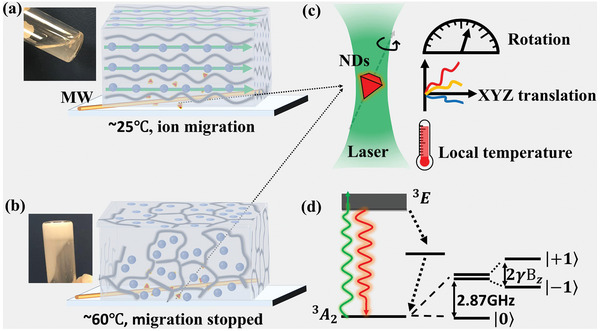
Schematic of ND based quantum sensing for ion‐conductive switch in thermo‐responsive hydrogel in situ. Geometrical configuration of home‐built electrochemical device for ODMR measurement of NDs in PNIPAM‐AM aqueous solution at a) ≈25 °C and b) ≈60 °C, corresponding to sol and gel state, respectively. c) The enlarged plot in (a) and (b), showing the optical and non‐invasive ND based quantum sensing for rotation, translation and local temperature. d) Structure of energy levels of NV centers.

As illustrated in **Figure** **6**a,b, NDs were pre‐introduced and randomly dispersed in the hydrogel, enabling that the NDs might occur in different phases or at the boundary after sol‐gel phase transition. The NDs rotation, translation and local temperature at different locations could be decoded from ODMR spectra of each ND (Figure 6c), measured as a decrease of fluorescence intensity when spin‐resonant microwave excitation was applied, because the spin excitation activated the nonfluorescent relaxation pathway (Figure 6d). In detail, for rotation and translation sensing, ODMR under applied magnetic field was recorded, while for local temperature sensing, no external magnetic field was applied for a better ODMR contrast. The ND translation was monitored by the 3D location of each ND.^[^ ^20a^ ^]^ Resonant frequencies of NV centers in NDs were obtained from the fitting result of each ODMR spectrum. The fluctuations of resonant frequencies suggested the rotation of NDs. The real time zero‐field splitting *D*(*t*) was calculated by averaging the resonant frequencies. Then the local temperature recorded by NDs was determined as

(1) $$T\;\left(t\right)=\;{\left(\frac{dD}{dT}\right)}^{-1}\left[D\left(t\right)-D_{0}\right]+T_{0}$$

where *dD*/*dT* represented the temperature susceptibility, and *D*_0_ was the referenced zero‐field splitting at the temperature of *T*_0_

By correlating the recorded information with phase change in hydrogel, the viscoelastic and thermodynamic properties determining the ion migration in the corresponding sol‐gel phase transition could be visualized.

### Measurement of Electrical Resistance

A Pt 100 was used to record the average temperature in hydrogel. A ceramic with the size of 1.3 mm × 1.1 mm × 0.9 mm was used as the substrate of the sensor tip. Temperature responsive Pt grating was printed on the surface of the ceramic (Figure S31a,b, Supporting Information). A resistance bridge circuit (Figure S31c, Supporting Information) was used to dynamically record the effective electrical resistance of hydrogel during phase transition. Two resistors with the resistance of 100 Ohm were used as R1 and R2. A resistor of 120 Ohm was used as R3. The power supply provides a constant voltage of 2 V. The output voltage between V1 and V2 was recorded by NIDAQ card to coordinate with the simultaneous quantum sensing measurement. The temperature coefficient of Pt 100 was calibrated in the silicone oil bath before usage (Figure S31d, Supporting Information).

### Statistical Analysis

The Ruman and ODMR spectra were normalized. The ODMR spectra was fitted and analyzed by multi‐peak Lorentzian function. No data were excluded as outliers. Average data values were reported as mean ± standard deviation of the mean. The sample sizes for the data in each figure were indicated in the figure legends. The data and figures were processed and plotted using Python and Origin 2021.

## Conflict of Interest

The authors declare no conflict of interest.

## Author Contributions

R.Q.D. performed data curation, methodology, validation, wrote the original draft; Z.L. performed investigation, data curation; G.L.Z. performed data curation; C.L. performed data curation; F.L. performed investigation; B.W performed funding acquisition, wrote, reviewed and edited the final manuscript.

## Supporting information



Supporting Information

Supplemental Video 1

Supplemental Video 2

Supplemental Video 3

## Data Availability

The data that support the findings of this study are available from the corresponding author upon reasonable request.

## References

[1] a) Z. Li , J. Guan , Expert Opin. Drug. Del. 2011, 8, 991; 10.1517/17425247.2011.581656 21564003

[2] O. H. Kwon , A. Kikuchi , M. Yamato , Y. Sakurai , T. Okano , J. Biomed. Mater. Res., Part A 2000, 50, 82. 10.1002/(sici)1097-4636(200004)50:1<82::aid-jbm12>3.0.co;2-7 10644967

[3] a) F. Mo , H. Li , Z. Pei , G. Liang , L. Ma , Q. Yang , D. Wang , Y. Huang , C. Zhi , Sci. Bull 2018, 63, 1077; 10.1016/j.scib.2018.06.019 36755460

[4] a) M. Li , Z. Chen , J. Polym. Sci. 2021, 59, 2230;

[5] a) C. Yang , Z. Suo , Nat. Rev. Mater. 2018, 3, 125;

[6] N.‐H. Cao‐Luu , Q.‐T. Pham , Z.‐H. Yao , F.‐M. Wang , C.‐S. Chern , J. Colloid Interface Sci. 2019, 536, 536. 30388531 10.1016/j.jcis.2018.10.091

[7] P. J. Flory , J. Rehner Jr , Ann. N. Y. Acad. Sci. 1943, 44, 419.

[8] a) Y. Gao , J. Yang , Y. Ding , X. Ye , J. Phys. Chem. B 2014, 118, 9460; 25029067 10.1021/jp503834c

[9] D. T. Murray , M. Kato , Y. Lin , K. R. Thurber , I. Hung , S. L. McKnight , R. Tycko , Cell 2017, 171, 615. 28942918 10.1016/j.cell.2017.08.048 PMC5650524

[10] a) C. Wu , S. Zhou , Macromolecules 1995, 28, 8381;

[11] H. Dai , Q. Chen , H. Qin , Y. Guan , D. Shen , Y. Hua , Y. Tang , J. Xu , Macromolecules 2006, 39, 6584.

[12] a) G. Petrini , G. Tomagra , E. Bernardi , E. Moreva , P. Traina , A. Marcantoni , F. Picollo , K. Kvaková , P. Cígler , I. P. Degiovanni , Adv. Sci. 2022, 9, 2202014; 10.1002/advs.202202014 PMC9534962 35876403

[13] a) G. Balasubramanian , P. Neumann , D. Twitchen , M. Markham , R. Kolesov , N. Mizuochi , J. Isoya , J. Achard , J. Beck , J. Tissler , V. Jacques , P. R. Hemmer , F. Jelezko , J. Wrachtrup , Nat. Mater. 2009, 8, 383; 19349970 10.1038/nmat2420

[14] Y. V. Pleskov , Russ. J. Electrochem. 2002, 38, 1275.

[15] J. R. Olson , R. O. Pohl , J. W. Vandersande , A. Zoltan , T. R. Anthony , W. F. Banholzer , Phys. Rev. B 1993, 47, 14850. 10.1103/physrevb.47.14850 10005859

[16] C. Foy , L. Zhang , M. E. Trusheim , K. R. Bagnall , D. R. Englund , ACS Appl. Mater. Interfaces 2020, 12, 26525. 32321237 10.1021/acsami.0c01545

[17] a) D. A. Simpson , E. Morrisroe , J. M. McCoey , A. H. Lombard , D. C. Mendis , F. Treussart , L. T. Hall , S. Petrou , L. C. L. Hollenberg , ACS Nano 2017, 11, 12077; 29111670 10.1021/acsnano.7b04850

[18] G. Balasubramanian , I. Y. Chan , R. Kolesov , M. Al‐Hmoud , J. Tisler , S. Chang , C. Kim , A. Wojcik , P. R. Hemmer , A. Krueger , Nature 2008, 455, 648. 18833276 10.1038/nature07278

[19] F. Dolde , H. Fedder , M. W. Doherty , T. Nöbauer , F. Rempp , G. Balasubramanian , T. Wolf , F. Reinhard , L. C. Hollenberg , F. Jelezko , Nat. Phys. 2011, 7, 459.

[20] a) X. Feng , W.‐H. Leong , K. Xia , C.‐F. Liu , G.‐Q. Liu , T. Rendler , J. Wrachtrup , R.‐B. Liu , Q. Li , Nano Lett. 2021, 21, 3393; 33847115 10.1021/acs.nanolett.0c04864

[21] a) R. Dou , G. Zhu , W.‐H. Leong , X. Feng , Z. Li , C. Lin , S. Wang , Q. Li , Carbon 2023, 203, 534;

[22] a) K. Ambal , R. D. McMichael , Rev. Sci. Instrum. 2019, 90, 023907; 30831689 10.1063/1.5065515 PMC6619432

[23] T. Petit , L. Puskar , Diamond Relat. Mater. 2018, 89, 52.

[24] Q. Sun , Vib. Spectrosco. 2009, 51, 213.

