# Supplementary material for: Operando Decoding Ion‐Conductive Switch in Stimuli‐Responsive Hydrogel by Nanodiamond‐Based Quantum Sensing
Source: Adv Sci (Weinh). 2024 Sep 23;11(43):2406944. doi: 10.1002/advs.202406944 (PMC11578334; doi:10.1002/advs.202406944)
Supplement: Supplementary file 1 — Supporting Information [file ADVS-11-2406944-s004.pdf]

ADVANCED SCIENCE
Open Access

Supporting Information

for *Adv. Sci.*, DOI 10.1002/advs.202406944

Operando Decoding Ion-Conductive Switch in Stimuli-Responsive Hydrogel by Nanodiamond-Based Quantum Sensing

Ruqiang Dou, Zan Li, Guoli Zhu, Chao Lin, Frank X. Liu and Biao Wang**

Supplementary Information

for

Operando Decoding Ion-conductive Switch in Stimuli-responsive Hydrogel by Nanodiamond-based Quantum Sensing

Ruqiang Dou[a,b,*], Zan Li[b], Guoli Zhu[b], Chao Lin[b], Frank X. Liu[c], Biao Wang[a,d,*]

a.Research Institute of Interdisciplinary Sciences & School of Materials Science and Engineering, Dongguan University of Technology, Dongguan, 523808, China.

b.Department of Physics, The Chinese University of Hong Kong, Shatin, New Territories, Hong Kong, 999077, China

c. Department of Mechanical and Aerospace Engineering, Hong Kong University of Science and Technology, Clear Water Bay, Kowloon, Hong Kong, China

d. School of Physics and Sino-French Institute of Nuclear Engineering and Technology, Sun Yat-sen University, China

*Correspondence and requests for materials should be addressed to R.D (ruokin@link.cuhk.edu.hk) or B.W (wangbiao@mail.sysu.edu.cn)

*Address correspondence to Ruqiang Dou, ruokin@link.cuhk.edu.hk; Biao Wang, wangbiao@mail.sysu.edu.cn

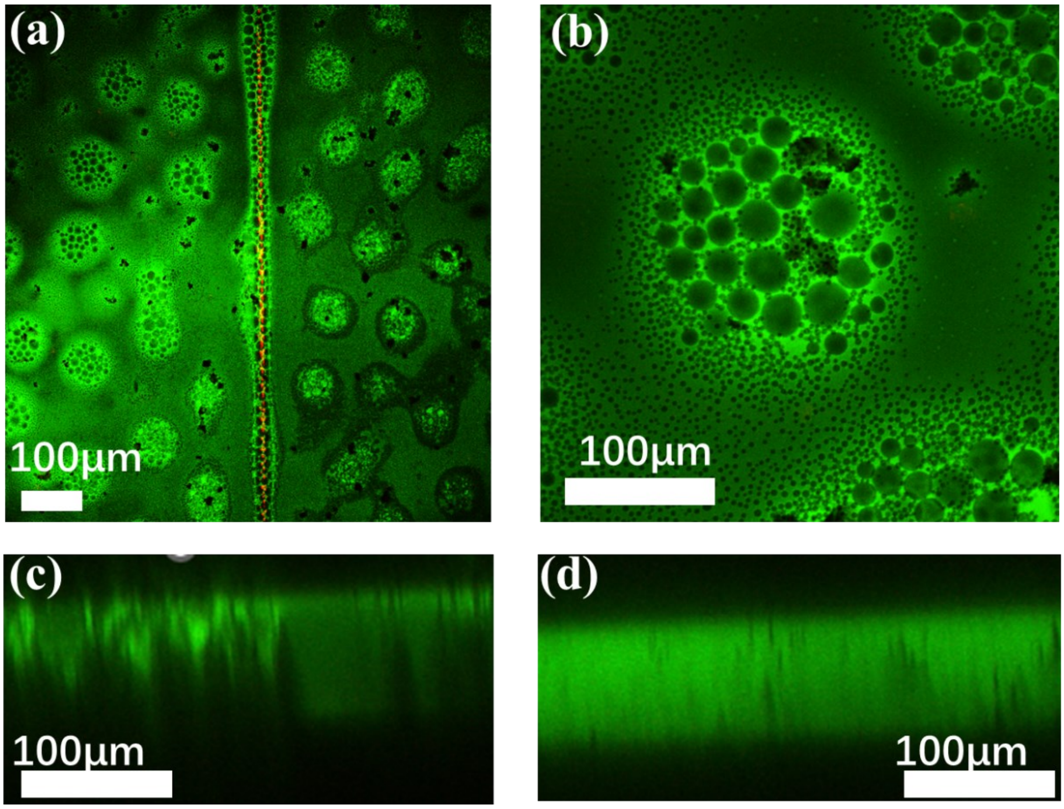

Figure S1. (a) Fluorescence image of FITC contained hydrogel at~60 ℃ taken by confocal microscope. Cross-linked phase separation accompanied with 'island' structure could be observed. (b) Enlarged image showing the fine structure in a small island. The fluorescence image of FITC contained hydrogel in z focus direction (vertical or perpendicular to cover glass) (c) at ~60℃ and (d) room temperature after cooling down.

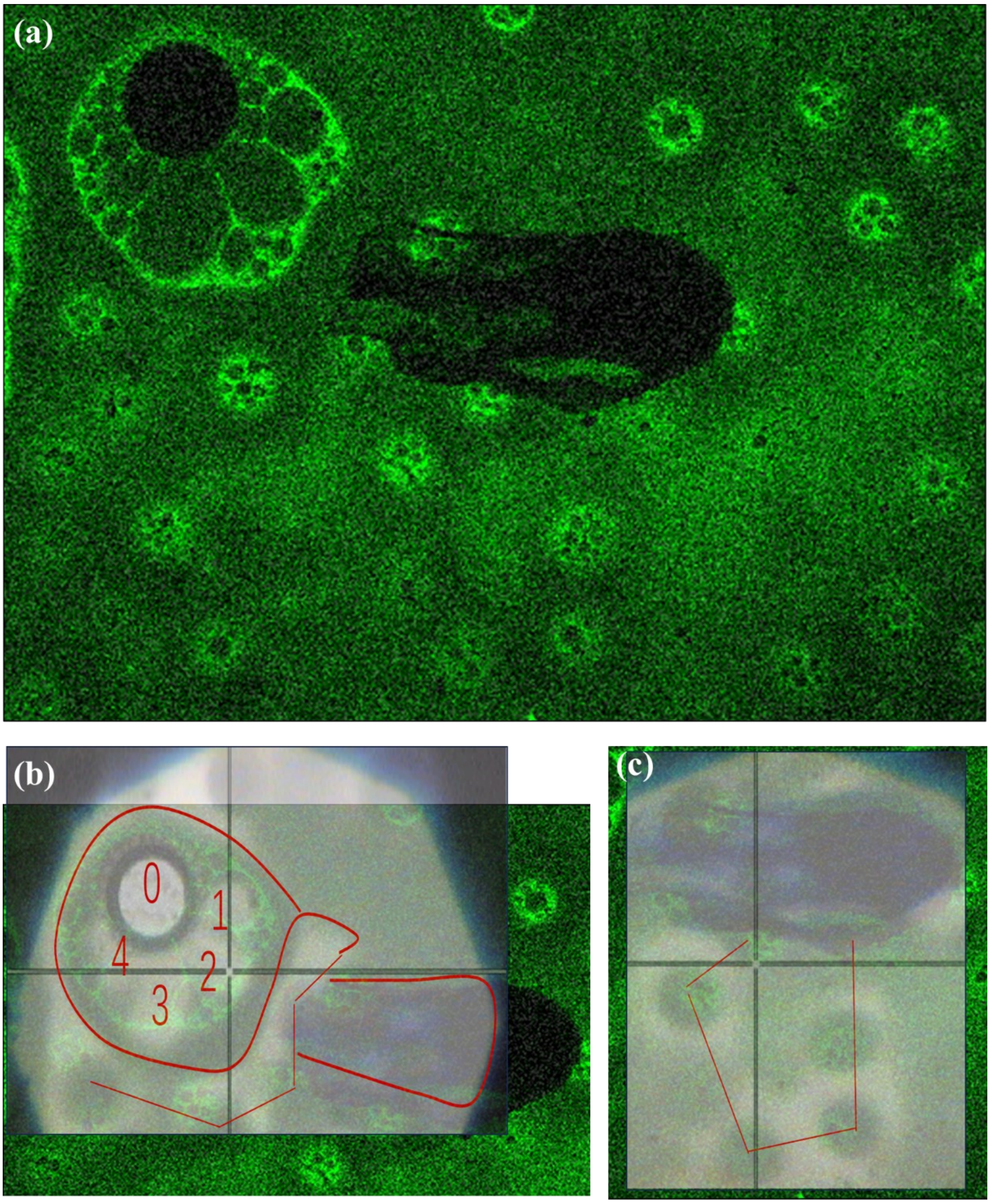

Figure S2. (a) Fluorescence image of FITC contained hydrogel after sol-gel phase transition with a black marker. (b-c) Merged figures of fluorescence image in confocal microscope and optical photos taken by camera equipped in Raman microscope.

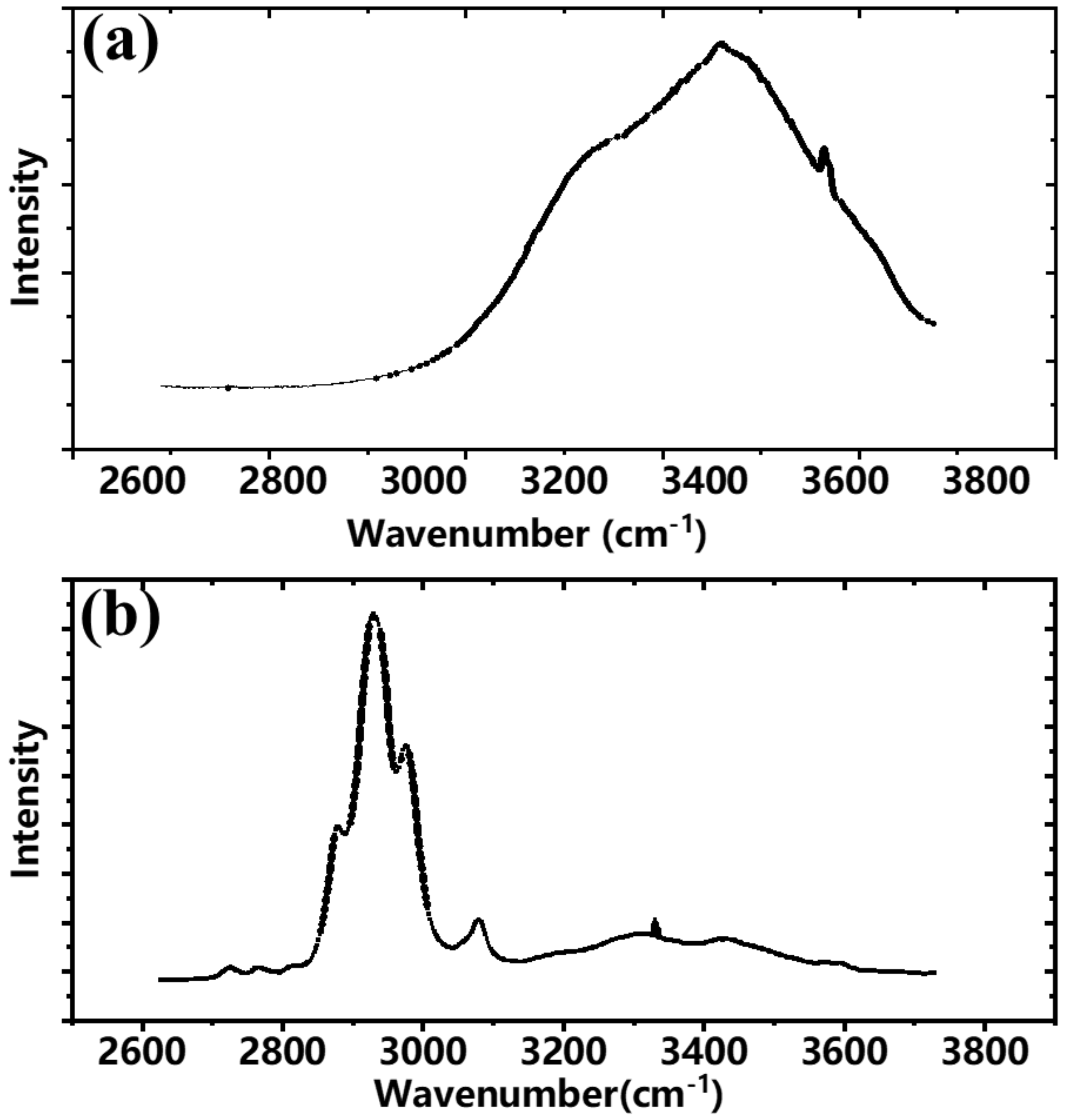

Figure S3. Raman spectra of (a) pure water and (b) dried hydrogel (PNIPAM-AM) powder.

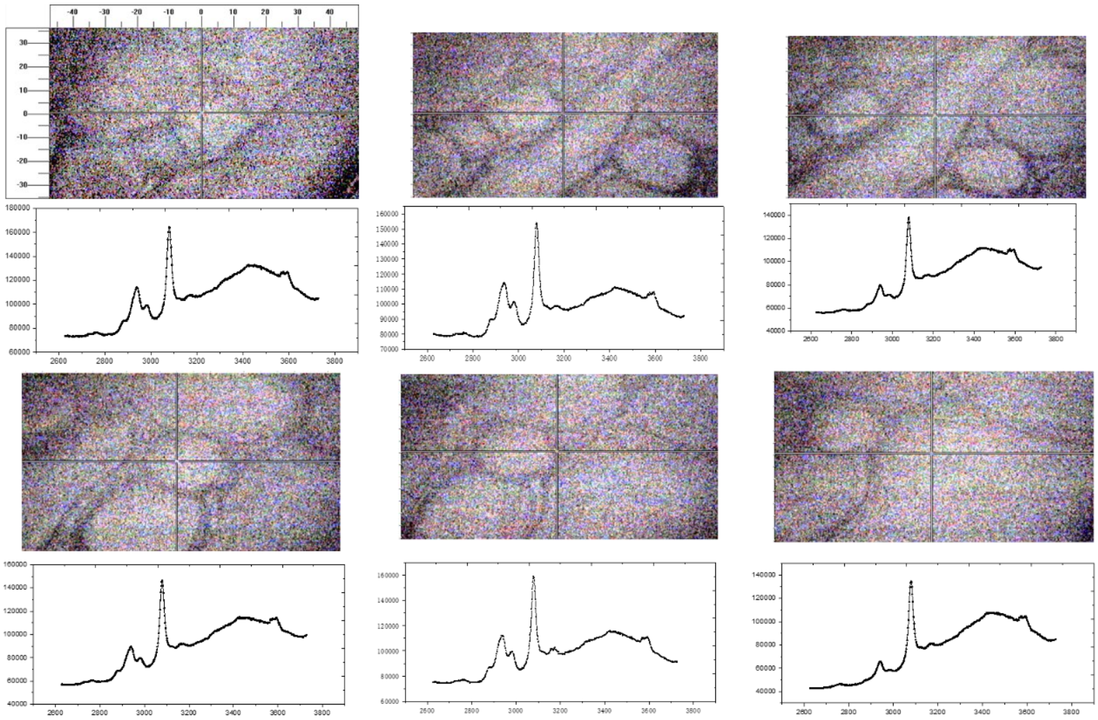

Figure S4. The different regions and corresponding Raman spectrum. The bright region represents water rich phase and the dark region represents water poor (polymer rich) phase.

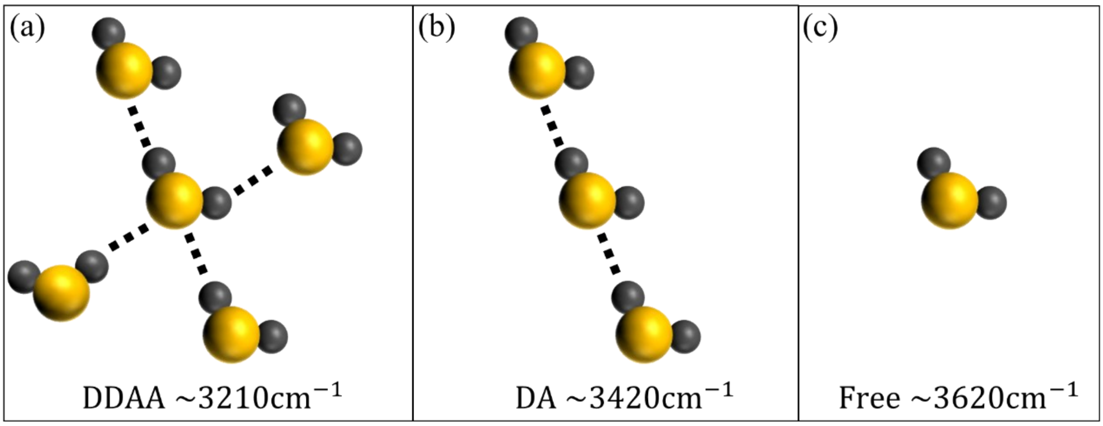

Figure S5. Hydrogen bonding configuration of water molecules. (a)DDAA: -OH stretching vibration with symmetrical HB regulation, that is, H_2O both donates and accepts two HBs to form a symmetrical tetrahedral structure. (b) DA: -OH stretching vibration with asymmetrical HB regulation, meaning that H_2O donates one and accepts one HB to form an asymmetrical line structure. (c) Free: free -OH (with no HB regulation) stretching vibration.

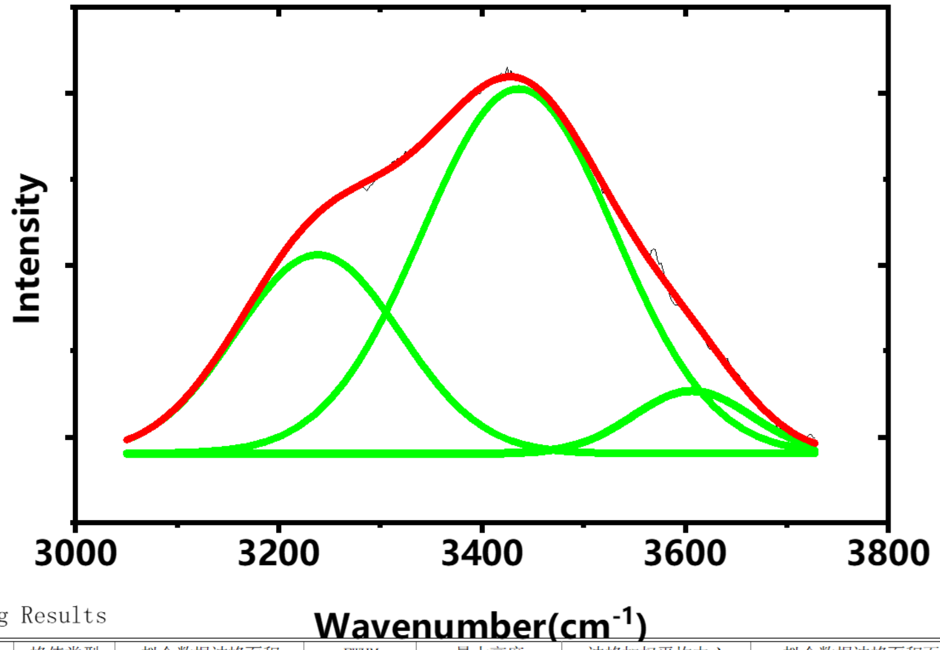

Fitting Results

波峰序号	峰值类型	拟合数据波峰面积	FWHM	最大高度	波峰加权平均中心	拟合数据波峰面积百分比
1	Gaussian	1.16231E7	191.0525	57747.59195	3238.52263	29.49479
2	Gaussian	2.51962E7	223.61641	105968.04015	3436.17173	63.93782
3	Gaussian	2588034.816	136.04551	18201.87789	3606.76845	6.56739

Figure S6. Raman spectrum and fitting result of hydrogel at room temperature.

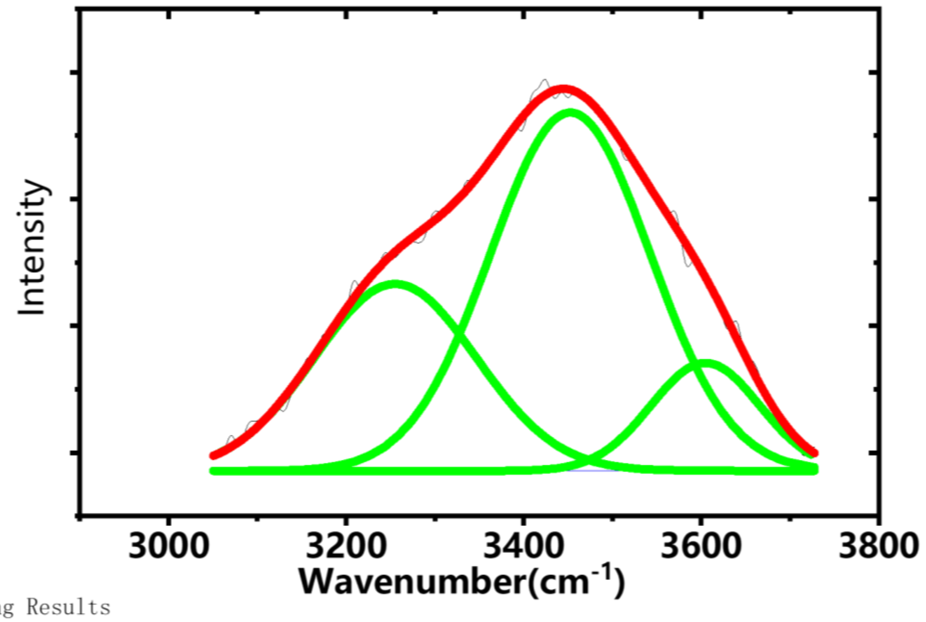

Fitting Results

波峰序号	峰值类型	拟合数据波峰面积	FWHM	最大高度	波峰加权平均中心	拟合数据波峰面积百分比
1	Gaussian	0.33269	214.7123	0.00147	3255.39071	30.32825
2	Gaussian	0.63654	211.83762	0.00283	3452.55084	58.02793
3	Gaussian	0.12773	144.21531	8.51112E-4	3604.61029	11.64382

Figure S7. Raman spectrum and fitting result of hydrogel at ~ 60℃ after sol-gel phase transition, representing the water rich phase.

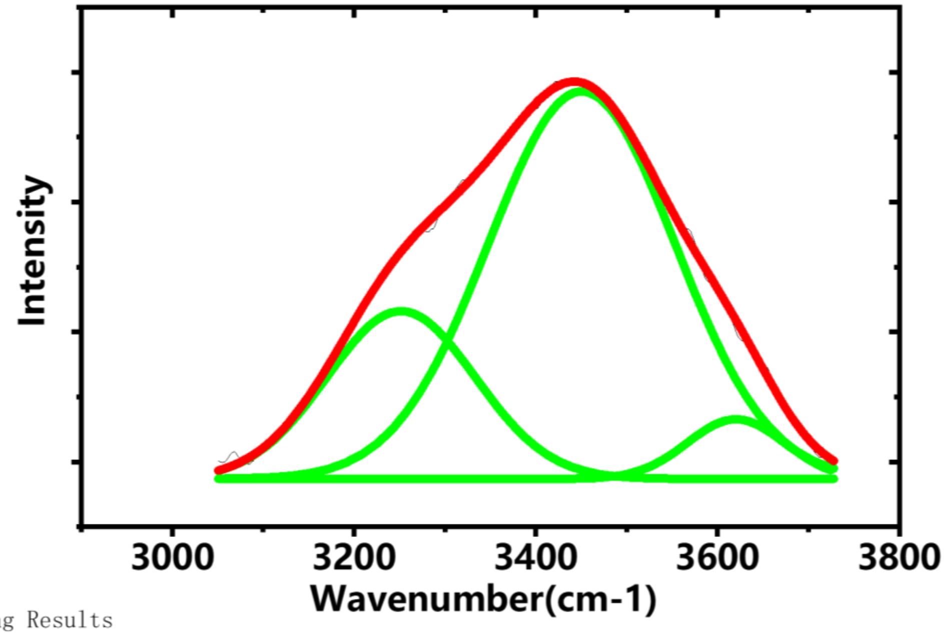

Fitting Results

波峰序号	峰值类型	拟合数据波峰面积	FWHM	最大高度	波峰加权平均中心	拟合数据波峰面积百分比
1	Gaussian	0.26233	192.34612	0.00129	3251.66177	24.10494
2	Gaussian	0.76616	242.17238	0.00298	3450.54217	70.3999
3	Gaussian	0.0598	125.33918	4.58163E-4	3620.16392	5.49516

Figure S8. Raman spectrum and fitting result of hydrogel at ~ 60℃ after sol-gel phase transition, representing the water poor phase.

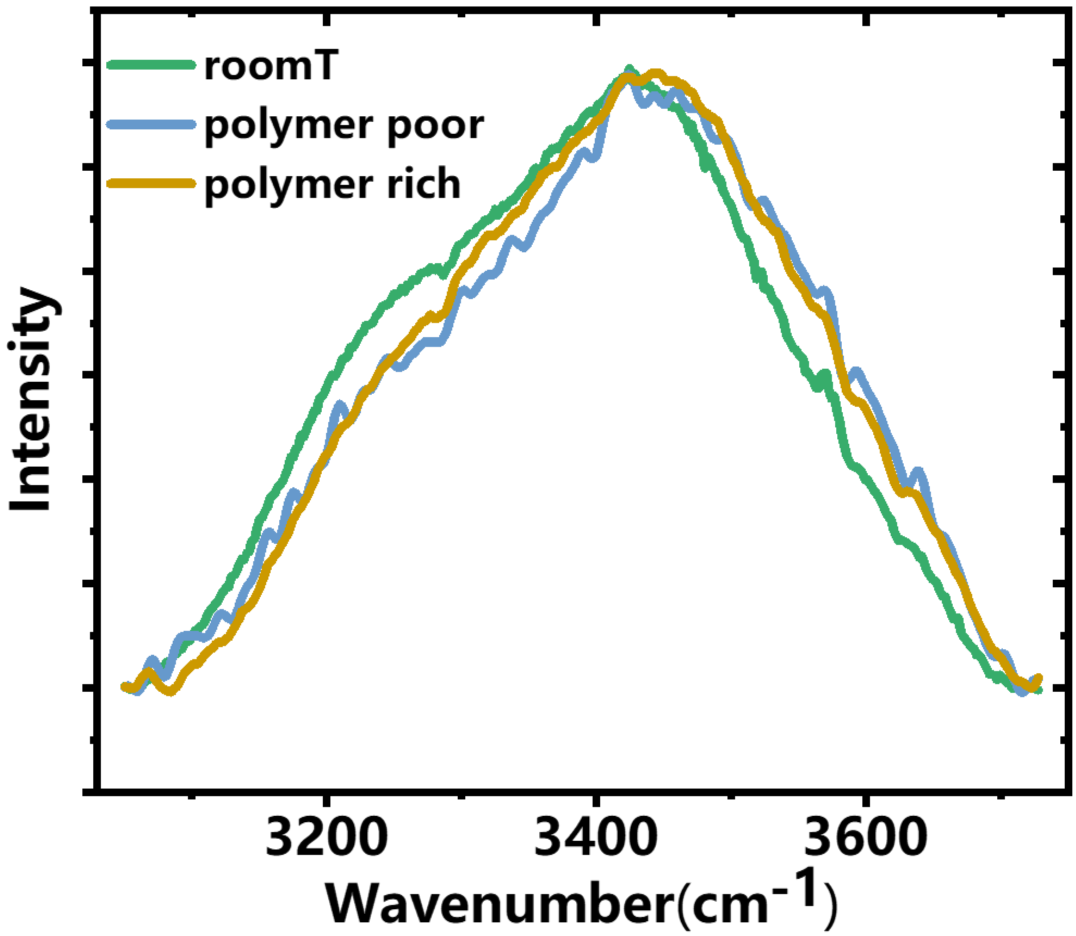

Figure S9. Normalized Raman spectra of hydrogel at room temperature and ~60℃ in water rich and poor phases for different phonon spectra analysis.

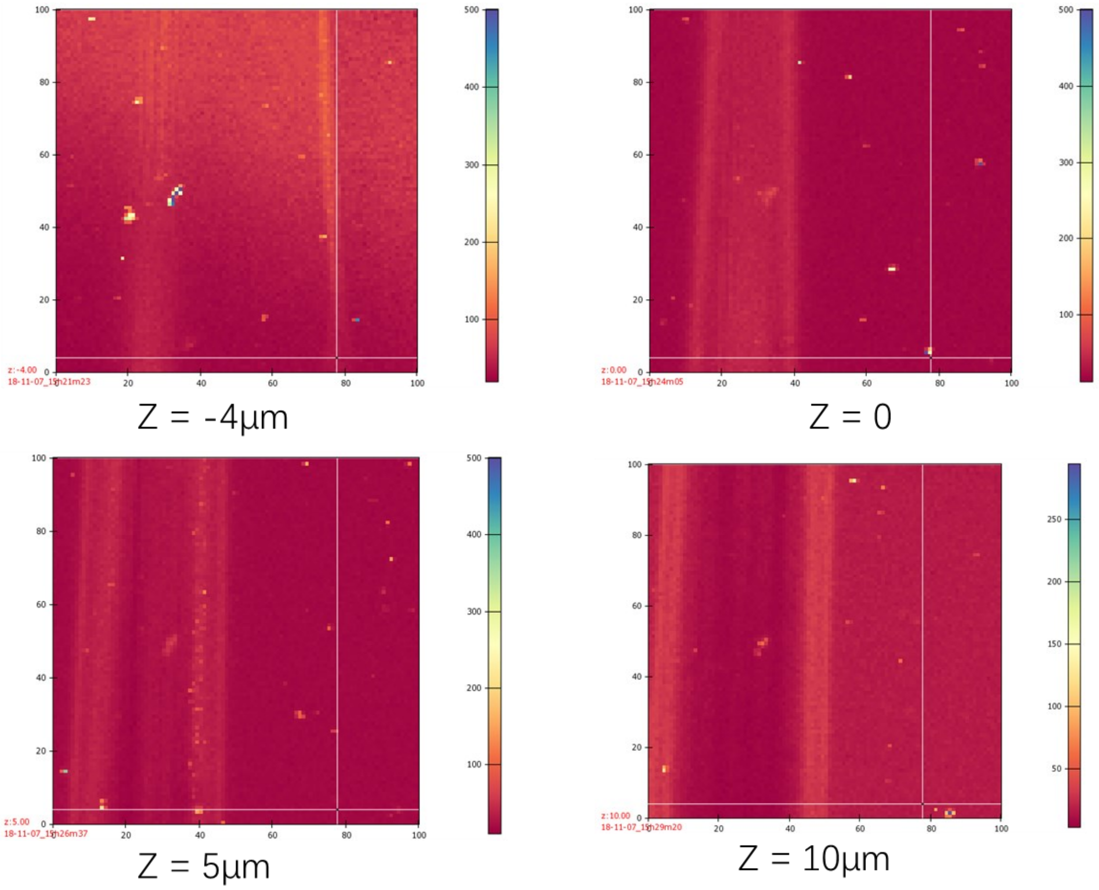

Figure S10. ND distribution in hydrogel at different focus. The bright points represent NDs, suggesting that NDs randomly distribute in the hydrogel at room temperature. Only several NDs could be found in the field of view, thus the hydrogen bonding interaction was dominated between water molecules and polymer chains. The contribution from the -OH group on the ND surface was small.

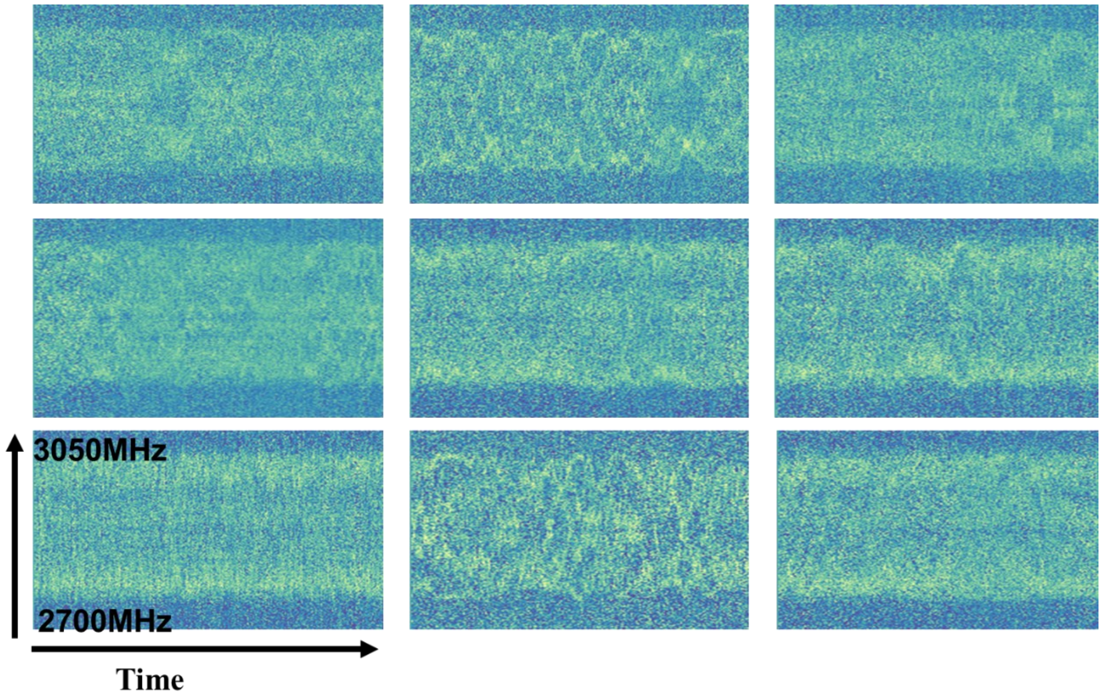

Figure S11. ODMR spectra of several examples of ND in hydrogel at room temperature. The NDs could be tracked during the entire duration time of 240s, while the eight ODMR peaks due to magnetic splitting were mixed together, suggesting the occurrence of considerable rotation.

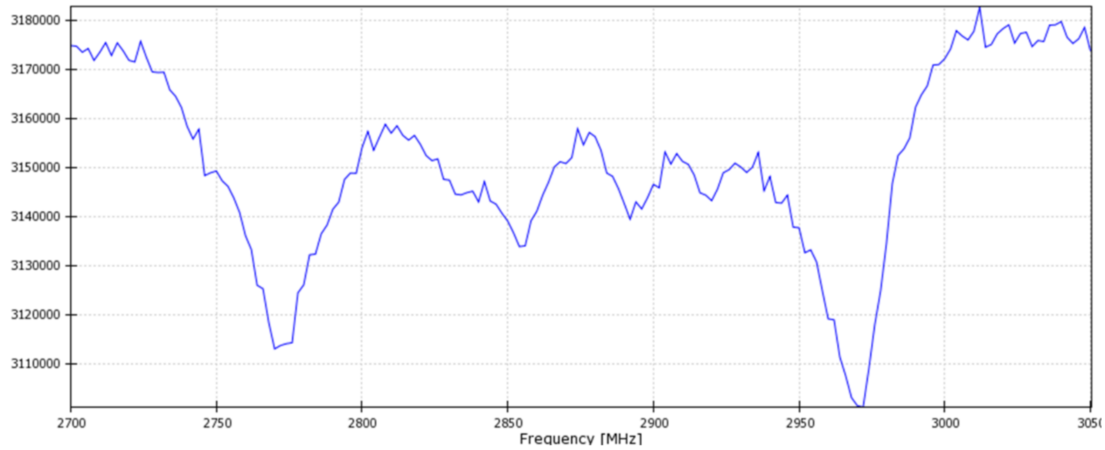

Figure S12. An example of ODMR spectrum of the ND in Figure 3 in main text. The peak splitting was due to applied magnetic field.

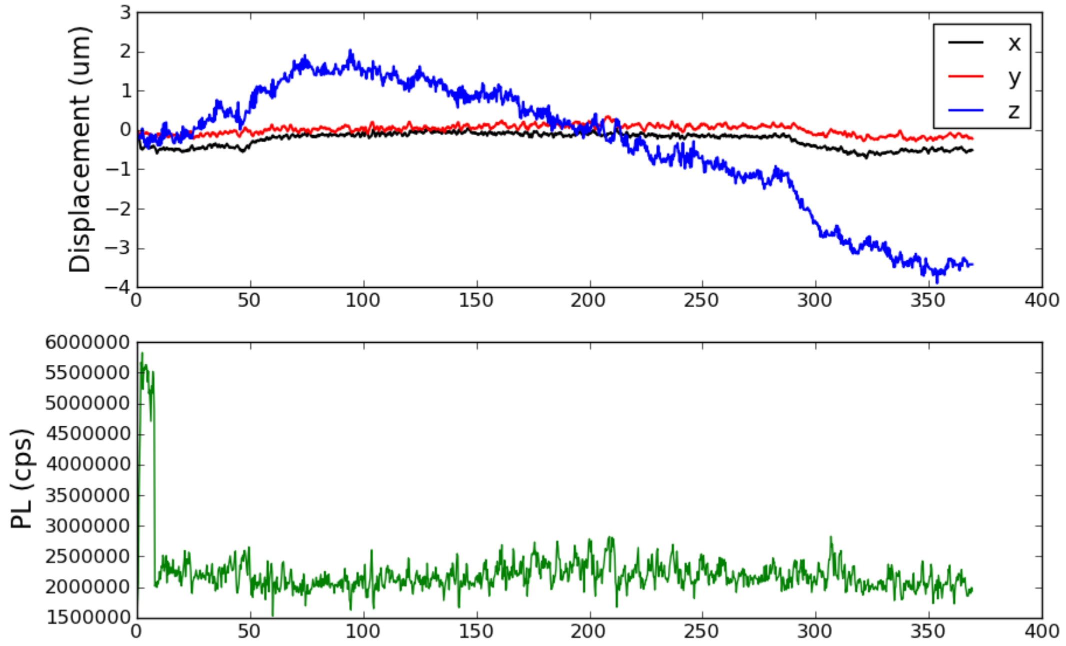

Figure S13. Displacement in *xyz* direction of an ND attached on the cover glass (without hydrogel) at ~60℃. Due to the cover glass was fixed on a PCB board, the *xy* direction was confined and *z* direction was not confined for focus adjustment. So considerable displacement in *z* direction due to thermal drift was usually observed.

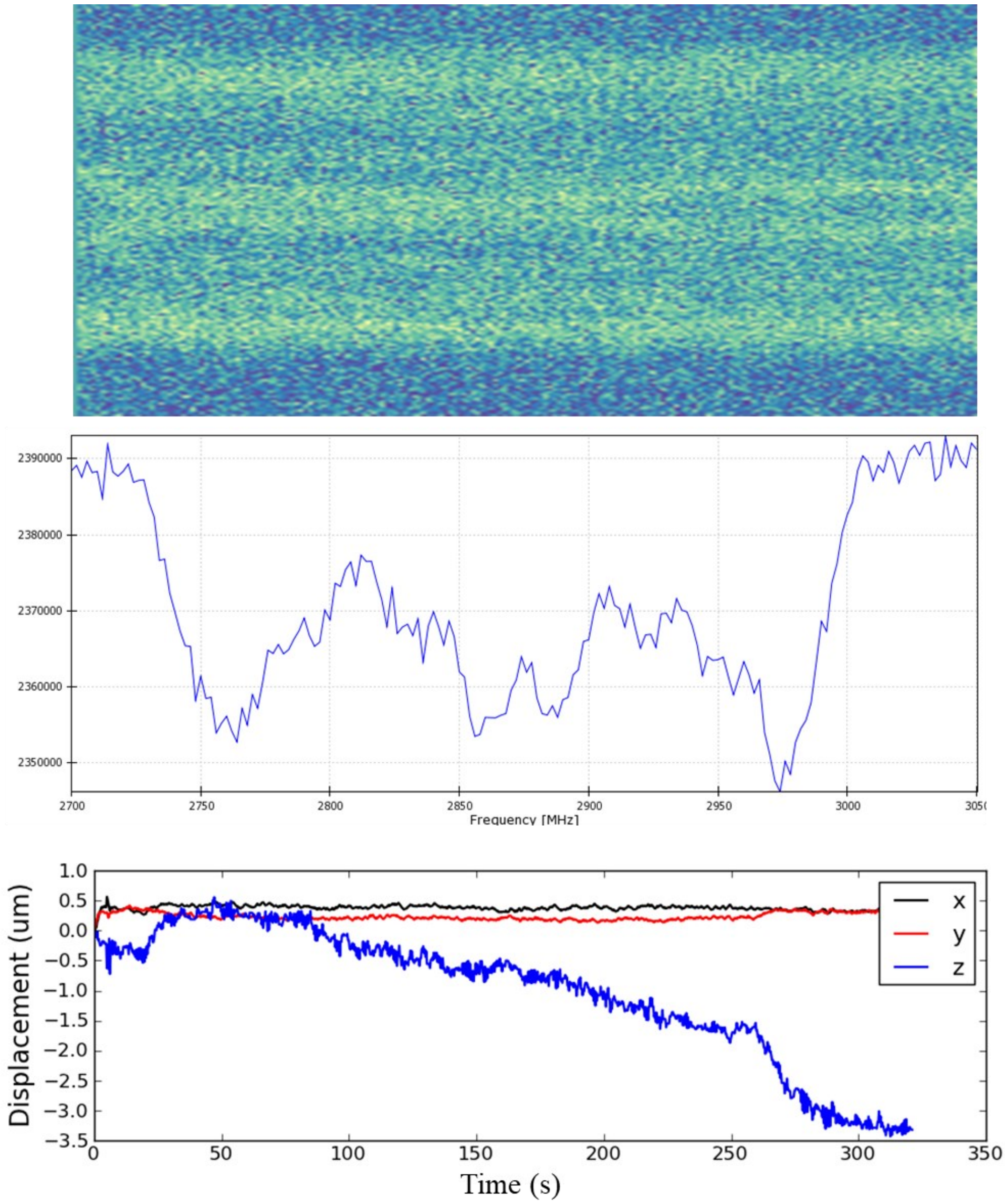

Figure S14. Another example showing ND rotation and translation in water poor phase. (a) Time dependent ODMR spectra under applied magnetic field with an example of ODMR spectrum shown in (b). The corresponding displacement in *xyz* direction. Little drift could be observed in *xy* direction. The enlarged drift in *z* direction came from thermal drift.

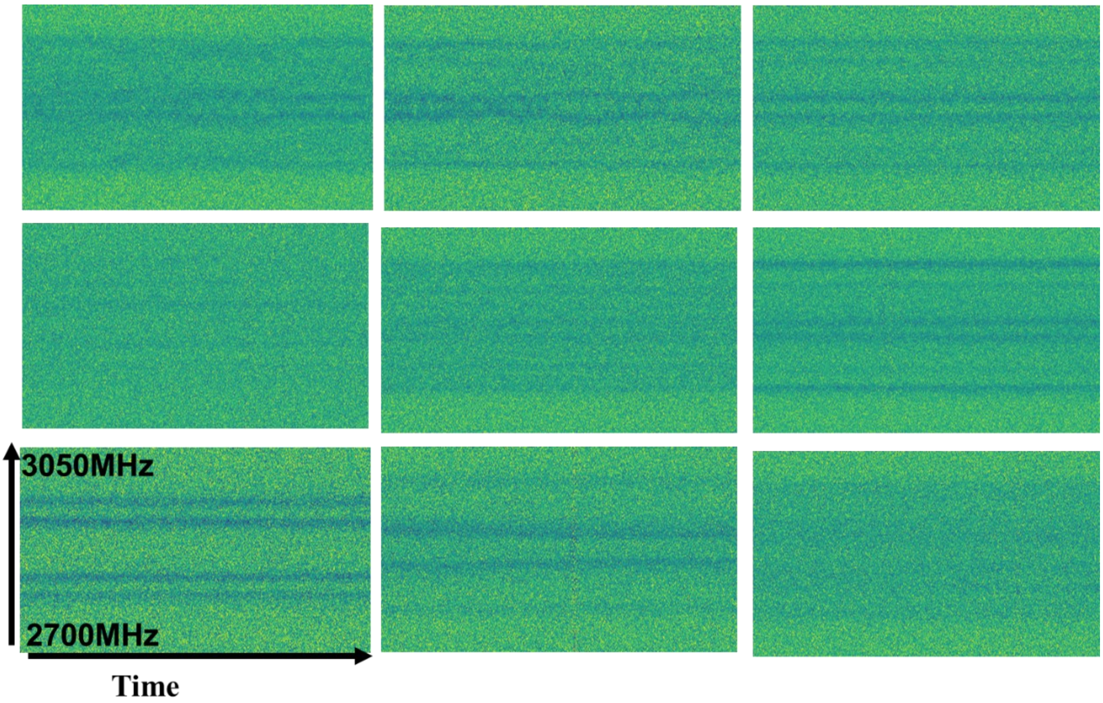

Figure S15. ODMR spectra of several examples of ND, located on the boundary of water rich and poor phases in hydrogel after sol-gel transition at ~60℃. The NDs could be tracked during the entire duration time of 300s. The resonance frequencies had a mild fluctuation, suggesting that the NDs at the interface of the two phases had small or even little rotation.

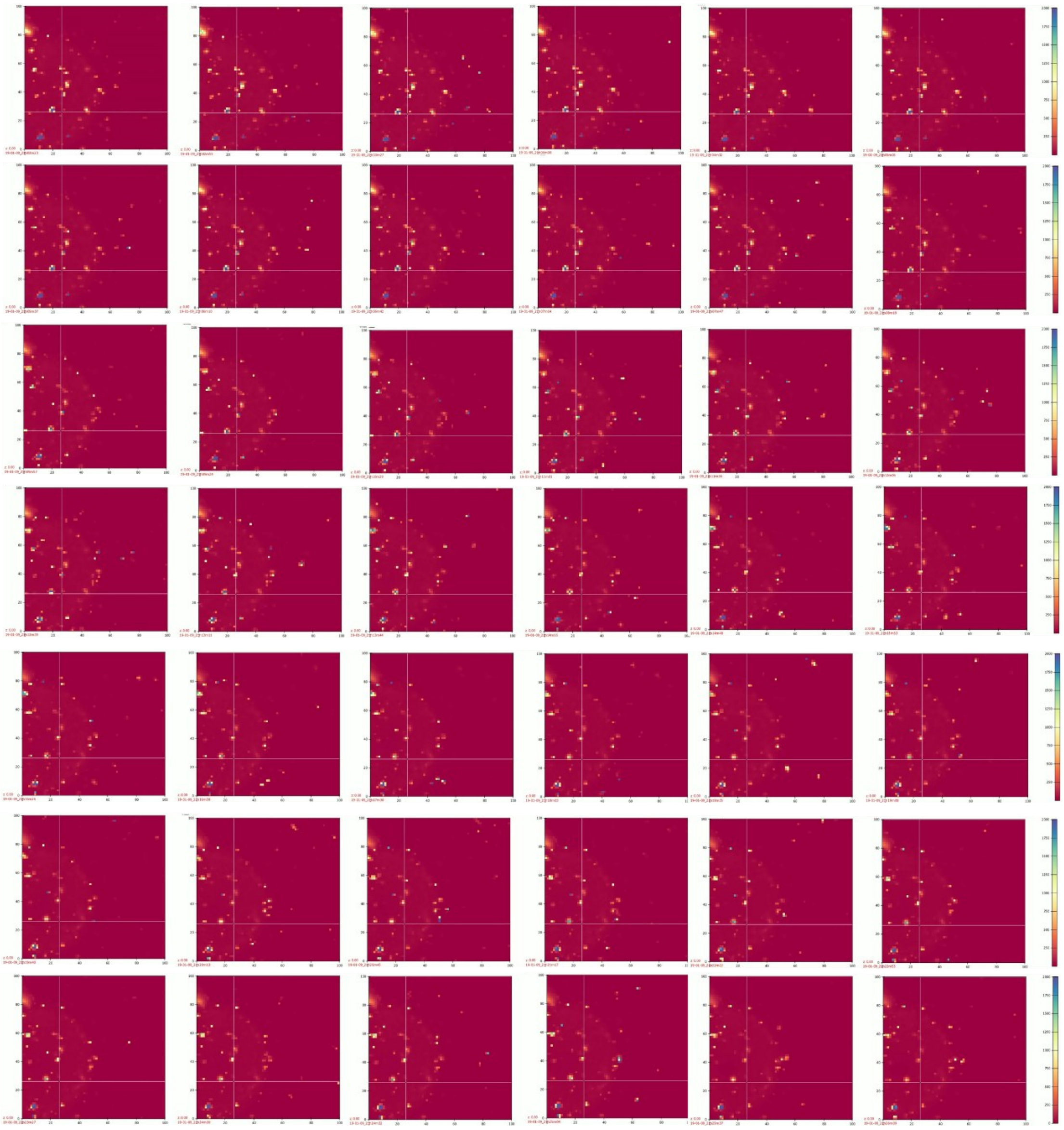

Figure S16. Time dependent ND distribution in different phase after sol-gel transition at ~60 ℃ with the phase separation shown in Figure 4(g) in main text. The duration time was ~24 min with the merged video could be found in supplementary video S2.

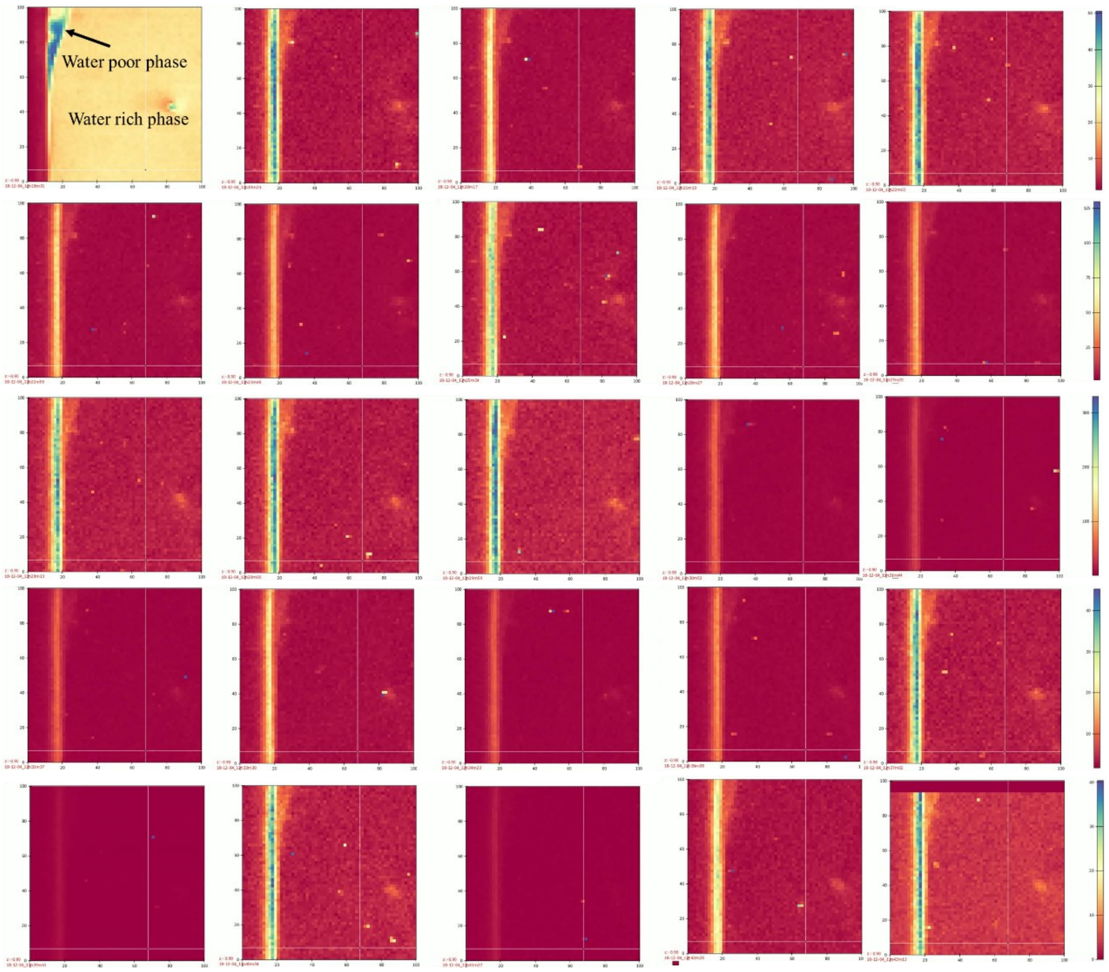

Figure S17. Time dependent ND distribution in water rich phase after sol-gel transition at ~60 ℃. The duration time was ~24 min. The image at left up conner presented the phase distribution. The NDs moved too fast to be tracked in water rich phase.

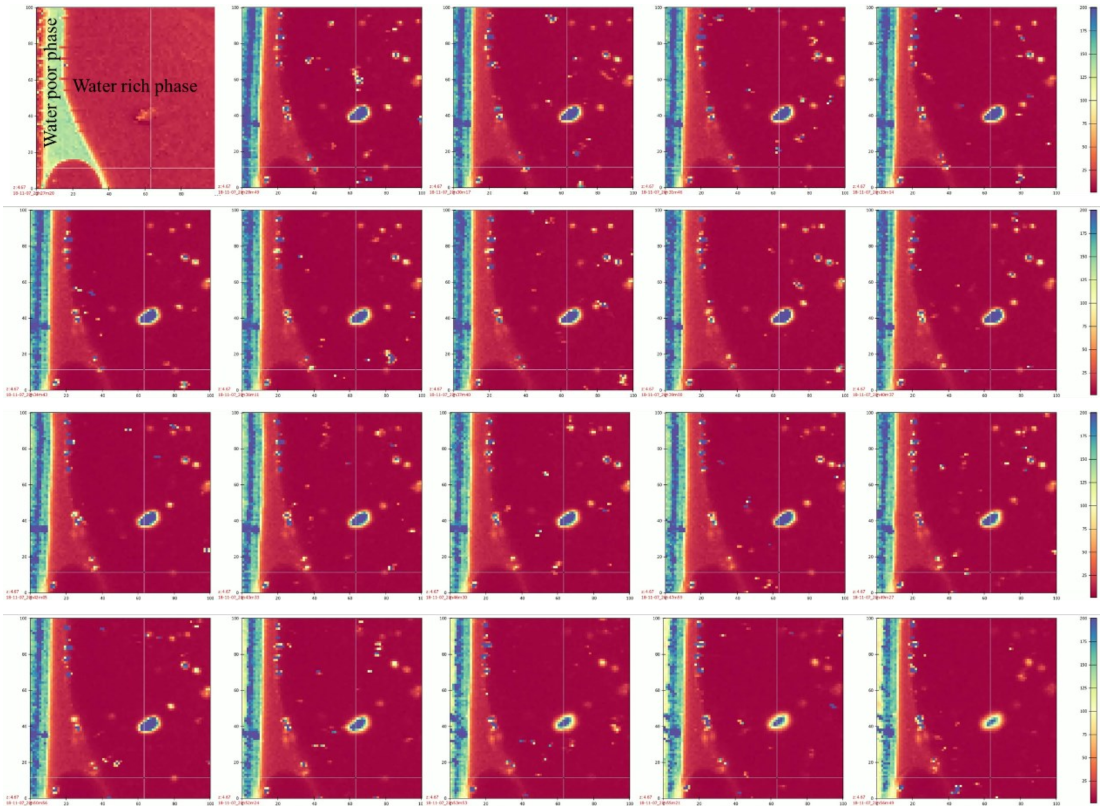

Figure S18. Time dependent ND distribution in hydrogel after sol-gel transition at ~60 ℃. The duration time was ~30 min. The image at left up corner showed the phase distribution. The NDs moved in the water rich phase. They might approach to the water poor phase, but could not break the interface of the two phases and transferred into the water poor phase. This could be visualized by the video in supplementary video S3.

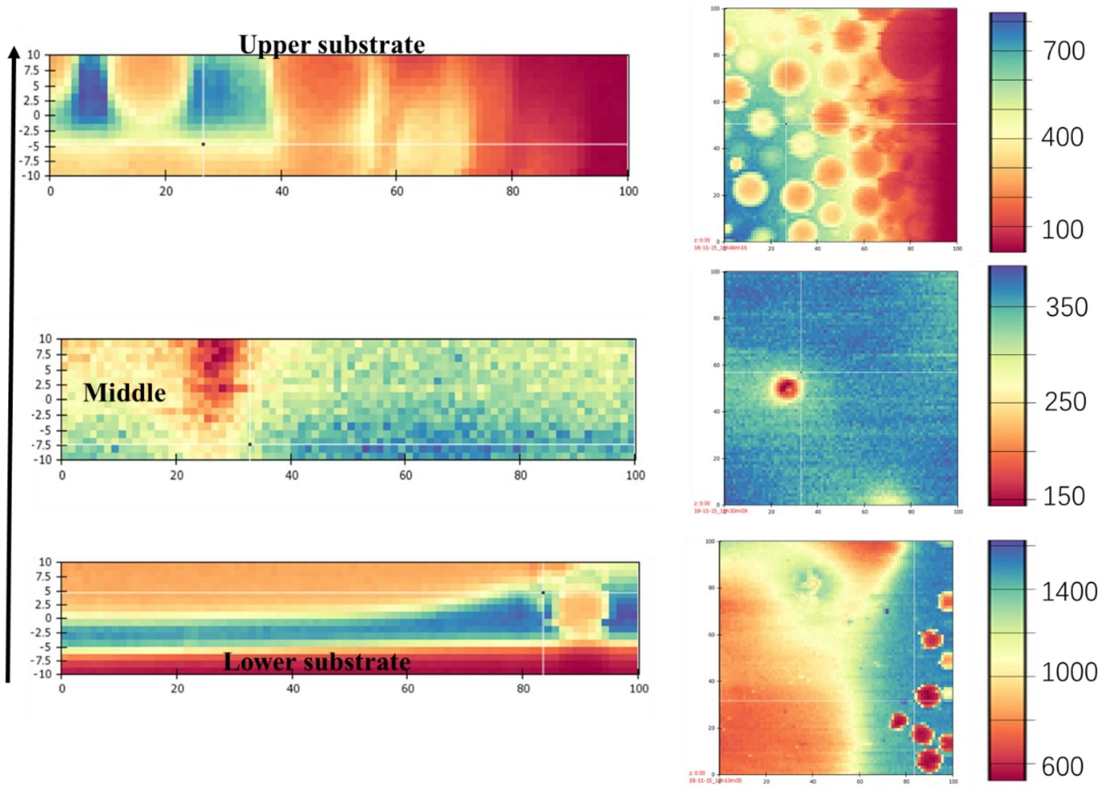

Figure S19. Phase distribution near lower and upper substrate and in between with the device geometrical configuration shown in Figure S28. The bright and less bright region corresponds to FITC rich and poor phase, respectively. Based on the correlation established in the main text, the water poor and rich phase were corresponded to the bright and less bright region. Water poor phase was prone to appear the substrate. Water rich phase distributes in between the lower and upper substrate and might be surrounded by water poor phase.

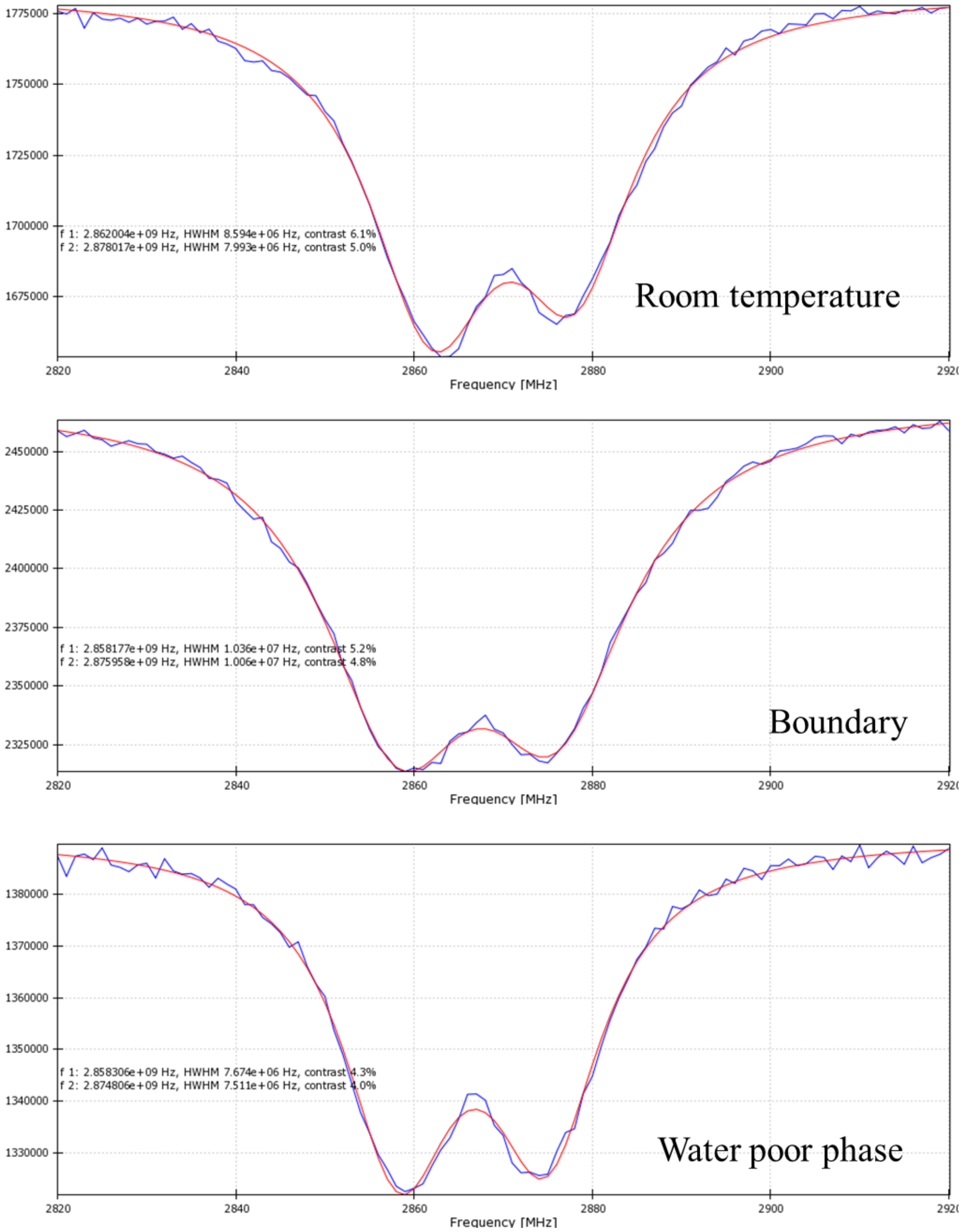

Figure S20. Examples of ODMR spectra in hydrogel at room temperature, in water poor phase and boundary of water poor/rich phase at high temperature after sol-gel transition for local temperature determination.

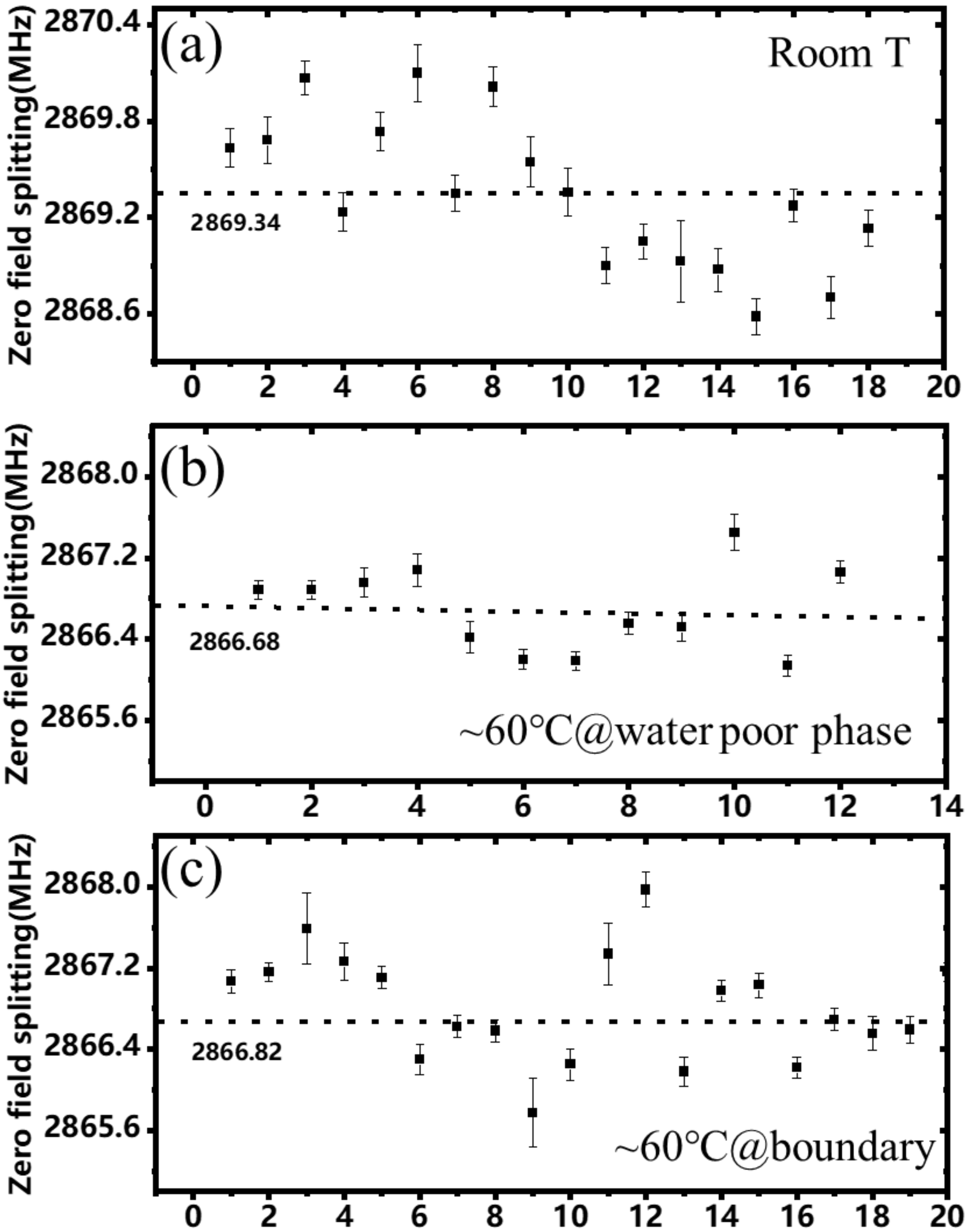

Figure S21. Local temperature fluctuations determined by individual NDs at room and high temperature. The scattering temperature points (a) in hydrogel at room temperature, (b) in water poor phase and (c) at the boundary of water poor/rich phase at ~60℃.

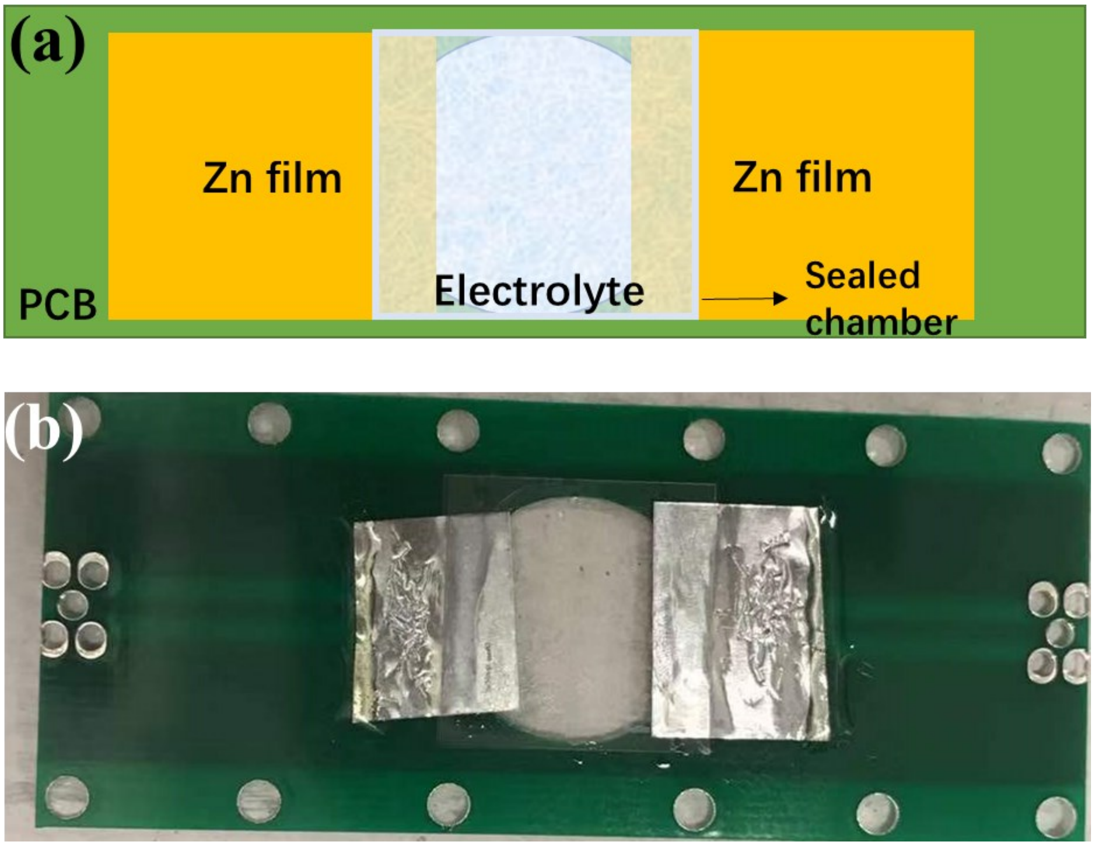

Figure S22. A specially designed resistor to test the concentration dependent ion conductive switch in thermo-responsive hydrogel. A (a) schematic and (b) photo of the resistor device.

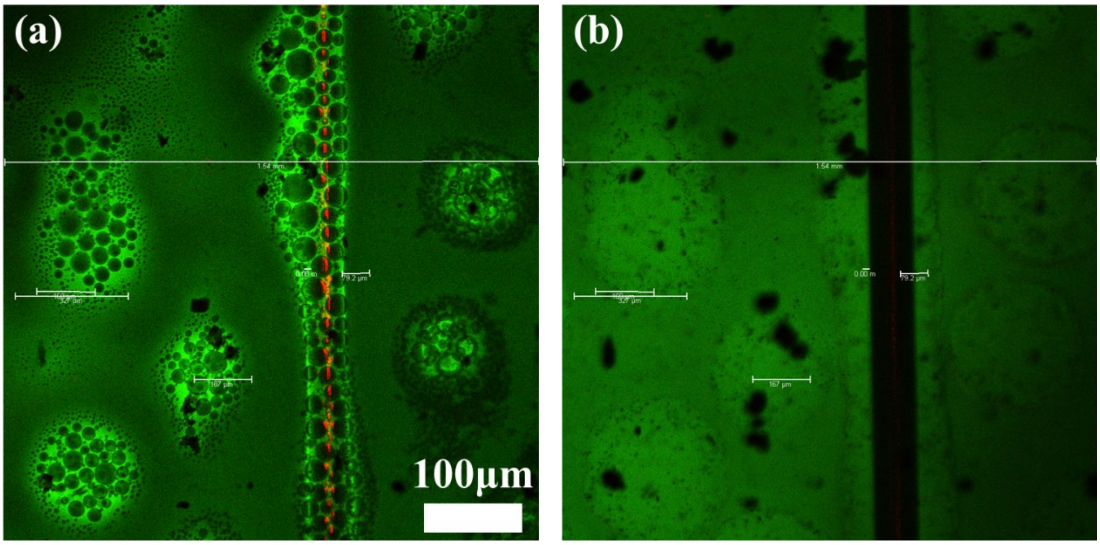

Figure S23. Fluorescence images of FITC contained hydrogel in a same imaging region at (a) gel state and (b) room temperature after cooling down. Usually, we could see the hydrogel transited from transparent sol state to opaque gel state when heating was applied. The hydrogel became transparent again when temperature was cooling down. The reversibility was good at macroscopic scale. In fact, the reversibility at nano-scale was not as good as that at macro-scale. As shown in Figure S23(b), a shell-like structure could be observed when temperature cooled down, corresponding to the gel 'island' at high temperature. This might be induced by inhomogeneous phase transition and lead to the resistance changing in heating and cooling cycles.

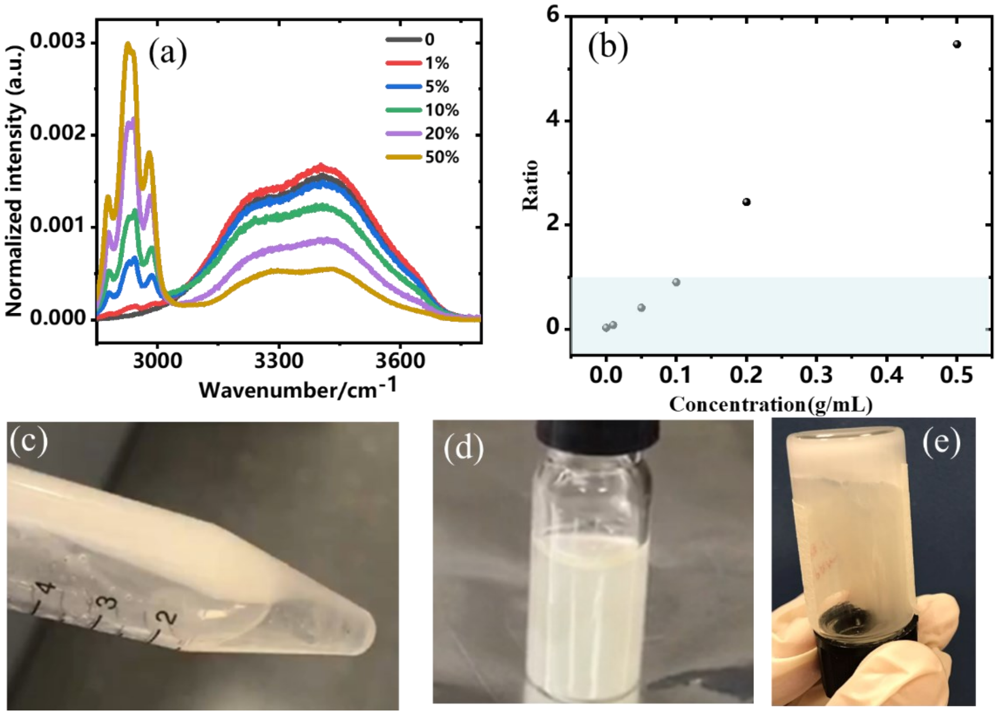

Figure S24. (a)Raman spectra of PNIPAM-AM hydrogel electrolyte at different concentrations. (b) The ratio as a function of concentration. The ratio defined as Raman intensity of polymer chains over that of water. Optical photos of PNIPAM-AM hydrogel after sol-gel transition with the concentration of (c) 0.05, (d) 0.08 and (e) 0.10 g/mL.

As presented in Figure S24(a), the Raman peak < 3000 cm^{-1} was attributed to polymer chains, and that in the range of 3300-3600 cm^{-1} was originated from water molecules. We defined a polymer ratio $Ratio = \frac{Raman\ intensity\ at\ 2926cm^{-1}}{Raman\ intensity\ at\ 3405cm^{-1}}$. The ratio as a function of polymer concentration could be found in Figure S24(b), a transition from Ratio < 1 to Ratio > 1 occurred at the polymer concentration of ~0.1 g/mL, indicating a polymer concentration controlled transition. As presented in Figure 6(a-b) in the main text, little free water separated from the hydrogel body after the sol-gel phase transition at the PNIPAM-AM hydrogel concentration of 0.2 g/mL. However when the polymer concentration decreased to 0.05 g/mL, free water could be easily observed post-transition (Figure S24c). Milk like opaque solution was observed when the polymer concentration was 0.08 g/mL (Figure S24d). Non-flowing opaque gel state started to occur when the polymer concentration was 0.1 g/mL (Figure S24e). Therefore, the concentration threshold for PNIPAM-AM ion conductive switch was ~0.08-0.1 g/mL, although the exact value was not obtained.

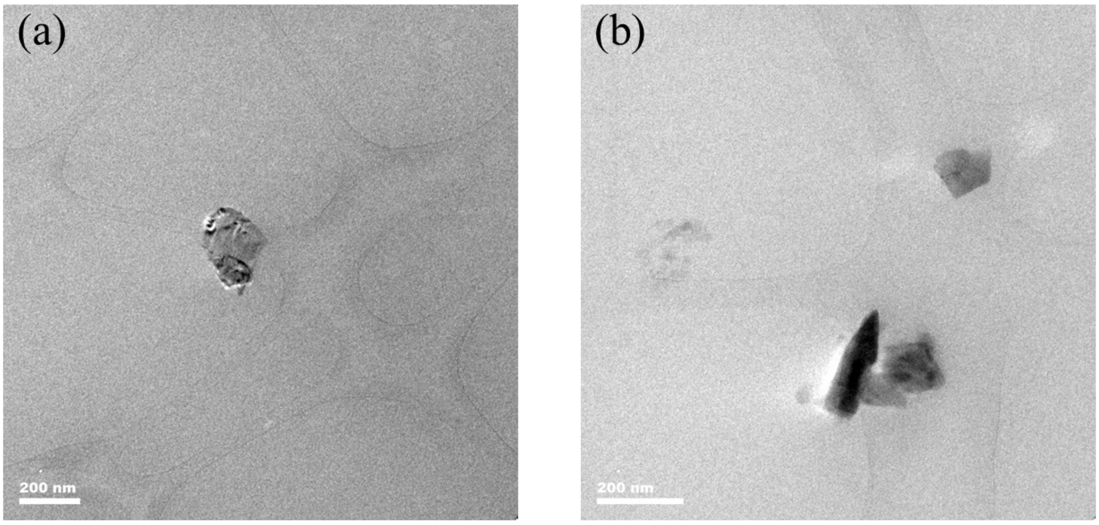

Figure S25. TEM images of nanodiamonds. The nanodimonds are irregular in shape with size ranging from a few tens to several hundred nanometers.

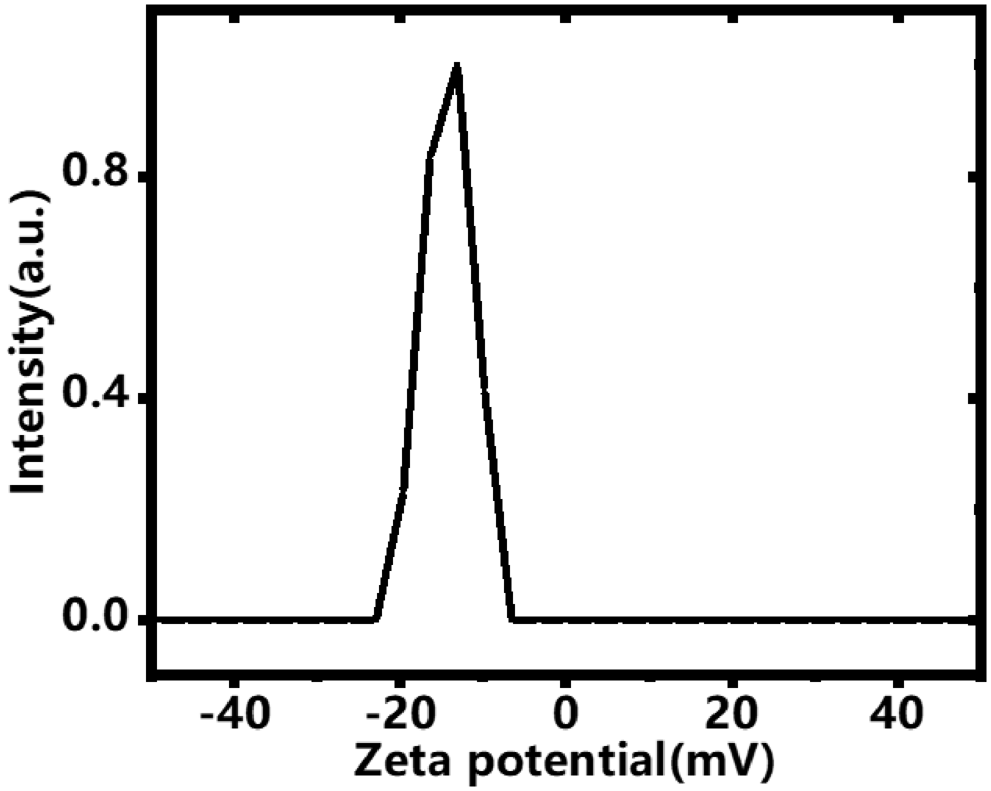

Figure S26. Zeta potential of nanodiamond sample. The negative zeta potential suggested that the nanodiamonds are negatively charged.

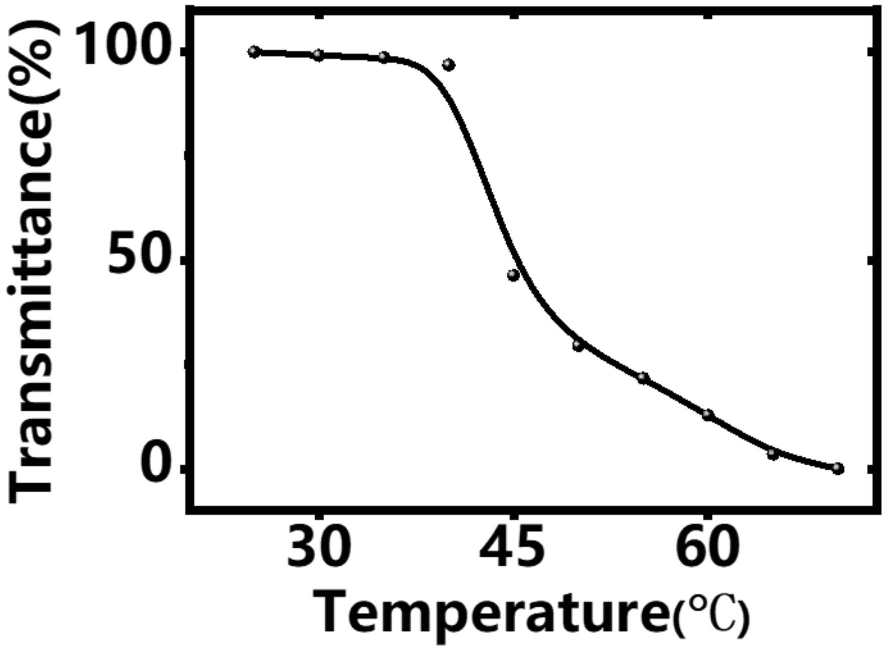

Figure S27. Transmittance of PNIPAM-AM hydrogel as a function of temperature, suggesting the LCST of ~45℃. One should note that LCST means the temperature of fastest transition from sol to gel state. Thus, the hydrogel does not fully become opaque gel state at LCST. The main reason to choose 60℃ as the test temperature was to ensure that almost all the hydrogel had become opaque gel state.

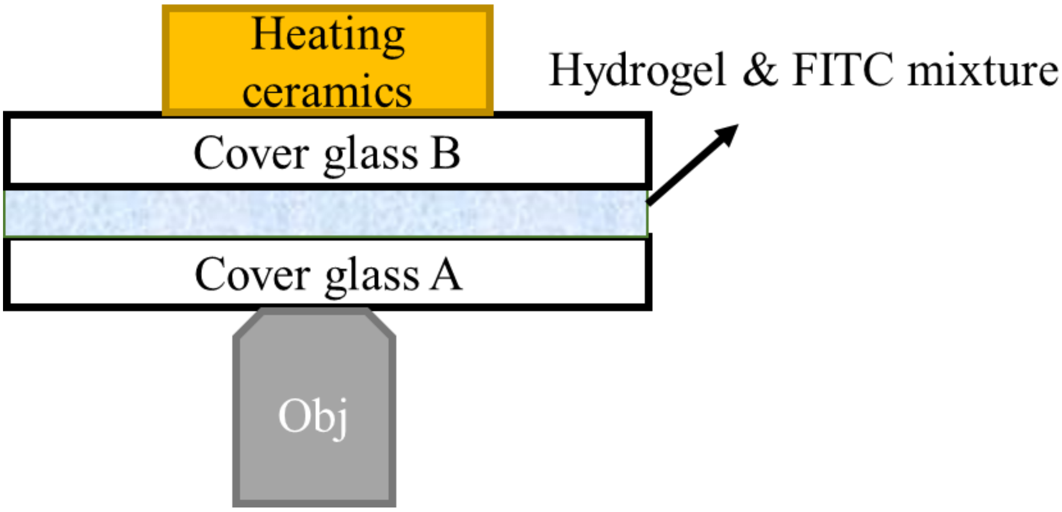

Figure S28. Geometrical configuration of the device used to *in situ* visualize the phase transition of hydrogel in confocal or Raman microscope, the hydrogel layer was ~100 μm. The edges were sealed with epoxy resin and the water loss due to evaporation could be negligible. In addition, hydrogel at 60℃ was almost fully on opaque gel state without free water separated from the hydrogel body, thus the loss on total water content was small and its influence on sol-gel transition/quantum sensing was negligible.

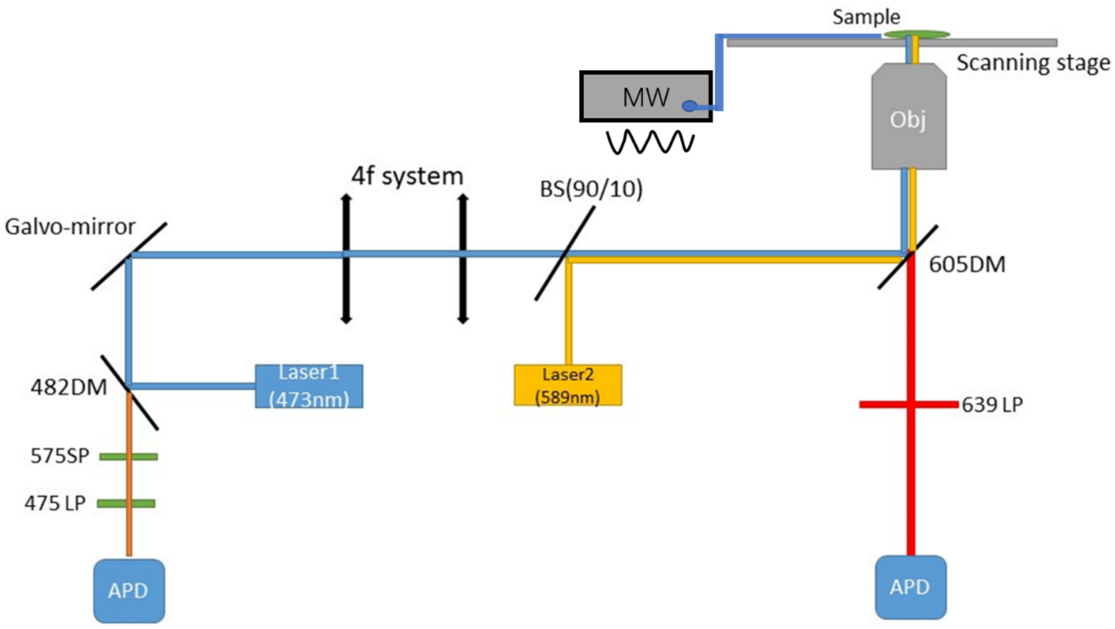

Figure S29. Setup of home-built confocal microscope for cw-ODMR measurement. APD: avalanche photo diode, LP: long pass filter, SP: short pass filter, DM: dic-mirror, BS: beam splitter, MW: microwave, Obj: objective lens.

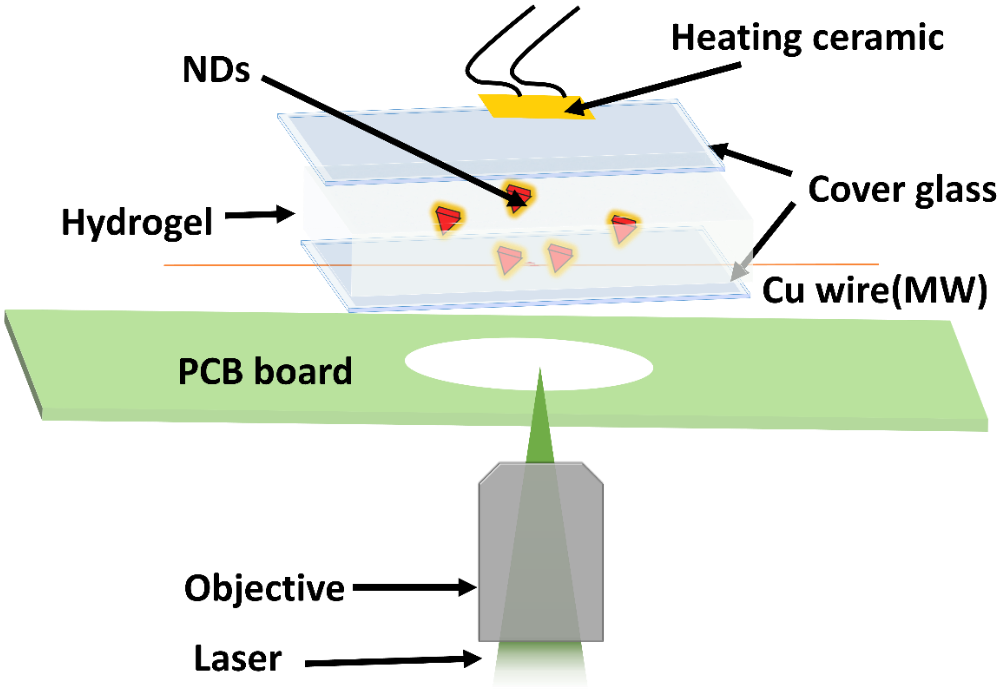

Figure S30. Schematic of the device for simultaneous ODMR measurement and thermo-responsive hydrogel phase transition

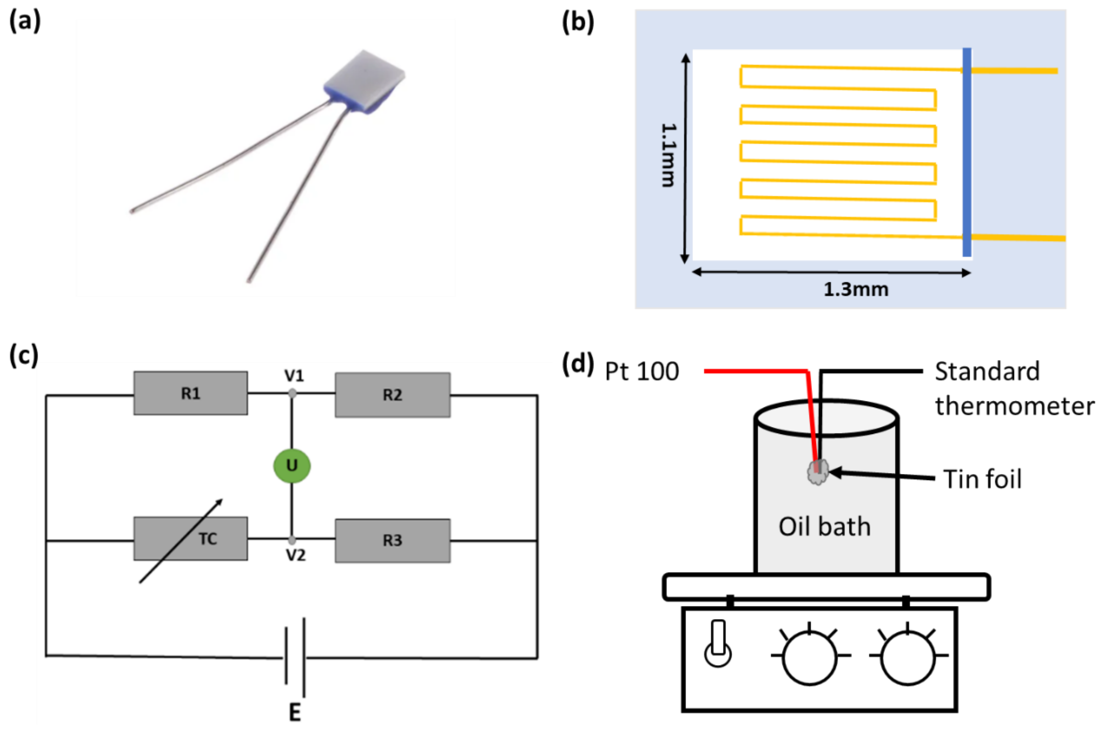

Figure S31. (a) A photo of a standard Pt 100 thermal resistor. (b) A schematic showing the Pt grating on the surface of the ceramic substrate. (c) Resistance bridge circuit to convert the temperature dependent resistance output to temperature dependent voltage output for NIDAQ card recording. (d) A diagram of heating method for the calibration of Pt 100.
